# Human genetic differentiation across the Strait of Gibraltar

**DOI:** 10.1186/1471-2148-10-237

**Published:** 2010-08-03

**Authors:** Mathias Currat, Estella S Poloni, Alicia Sanchez-Mazas

**Affiliations:** 1 Laboratory of Anthropology, Genetics and Peopling history (AGP), Department of Anthropology, University of Geneva, Switzerland

## Abstract

**Background:**

The Strait of Gibraltar is a crucial area in the settlement history of modern humans because it represents a possible connection between Africa and Europe. So far, genetic data were inconclusive about the fact that this strait constitutes a barrier to gene flow, as previous results were highly variable depending on the genetic locus studied. The present study evaluates the impact of the Gibraltar region in reducing gene flow between populations from North-Western Africa and South-Western Europe, by comparing formally various genetic loci. First, we compute several statistics of population differentiation. Then, we use an original simulation approach in order to infer the most probable evolutionary scenario for the settlement of the area, taking into account the effects of both demography and natural selection at some loci.

**Results:**

We show that the genetic patterns observed today in the region of the Strait of Gibraltar may reflect an ancient population genetic structure which has not been completely erased by more recent events such as Neolithic migrations. Moreover, the differences observed among the loci (i.e. a strong genetic boundary revealed by the Y-chromosome polymorphism and, at the other extreme, no genetic differentiation revealed by HLA-DRB1 variation) across the strait suggest specific evolutionary histories like sex-mediated migration and natural selection. By considering a model of balancing selection for HLA-DRB1, we here estimate a coefficient of selection of 2.2% for this locus (although weaker in Europe than in Africa), which is in line with what was estimated from synonymous versus non-synonymous substitution rates. Selection at this marker thus appears strong enough to leave a signature not only at the DNA level, but also at the population level where drift and migration processes were certainly relevant.

**Conclusions:**

Our multi-loci approach using both descriptive analyses and Bayesian inferences lead to better characterize the role of the Strait of Gibraltar in the evolution of modern humans. We show that gene flow across the Strait of Gibraltar occurred at relatively high rates since pre-Neolithic times and that natural selection and sex-bias migrations distorted the demographic signal at some specific loci of our genome.

## Background

Geneticists are often faced with the acute problem of disentangling the effects of natural selection and demographic history on the evolution of different polymorphisms [1,2]. Such effects may be confounding in a number of cases: directional selection generally leads to a loss of genetic diversity within populations, which can hardly be distinguished from an effect of rapid genetic drift; balancing selection maintains genetic variation, which is also expected in case of intensive gene flow between populations; unimodal mismatch distributions may result either from purifying selection or from demographic expansion; also, linkage disequilibrium may have multiple causes among which selection and genetic drift are both strong candidates [3,4].

Although recent studies on the genetic history of human populations focus on the analysis of non-coding (e.g. STRs [5]) or genome-wide (e.g. SNPs [6,7]) markers, allowing to get rid of selective effects acting on individual loci, data on classical and molecular polymorphisms related to either coding (e.g. blood groups, HLA) or specific (e.g. mtDNA) DNA regions have been widely used to reconstruct human history and thus need a specific attention. Hypotheses of natural selection have been proposed for many of those markers, e.g. ABO [8], Duffy [9] and other blood groups [10], mtDNA [11-13] and the Y chromosome [14]. However, the strongest evidence for selection is certainly found for the major histocompatibility complex (MHC) in humans, HLA [1,15-17]. This is explained by the crucial role played by class I and class II molecules in both cell-mediated and humoral immunity. Because HLA frequency distributions are often observed to deviate significantly from neutral expectations towards an excess of heterozygotes, it is generally assumed that HLA evolves under a pathogen-driven selection mechanism whereby HLA heterozygotes would have increased fitness compared to homozygotes in a pathogen-rich environment. However, other hypotheses have also been proposed like frequency-dependent selection conferring selective advantage to rare alleles to which pathogens would not have had time to adapt, as well as fluctuating selection depending on environmental changes over time and space (see [15], for a review). These different forms of balancing selection also explain why an excess of non-synonymous compared to synonymous substitutions are found at the peptide-binding regions of the HLA molecules.

Despite so many empirical evidences suggesting that HLA evolved under the influence of natural selection, it is not known whether selection has significantly affected the patterns of HLA genetic diversity worldwide, compared to the effects of genetic drift. HLA genetic distances are generally correlated with geographic distances worldwide [18] and at continental scales [19,20], indicating that natural selection did not remove the traces of human migrations throughout the world. In agreement with this hypothesis, Tiercy et al. [21] estimated a very low selective coefficient at the HLA-DRB1 locus (from 2.51 × 10^-4 ^to 6.28 × 10^-4^, with an average of 4.42 ± 0.76 × 10^-4^), although variation in effective population size between populations may actually lead to more heterogeneous estimates. Also, based on the hypothesis that non-coding regions are neutral, Meyer et al. [1] compared the amounts of HLA and nuclear STR genetic variation in a set of identical population samples to estimate the amplitude of the deviation due to balancing selection on HLA, and found no indication of significantly reduced differentiation at HLA loci compared to STRs.

While the results mentioned above indicate a weak effect of natural selection at population level on HLA loci, some HLA population studies have revealed unexpected patterns that are not easily explained by the history and demography of human populations alone, in particular at the DRB1 locus. Sanchez-Mazas [19] found a lack of genetic differentiation (non-significant *F_ST_*) between a number of African, European and West Asian populations for HLA-DRB1, but not for HLA-DPB1, which is a locus that is supposed to evolve almost neutrally [22]. In addition, previous analyses of HLA-DRB1 revealed an absence of genetic differentiation (low to non-significant *F_ST_*) between some populations located on both sides of the Western Mediterranean region, i.e. North-Western Africa (NWA) and South-Western Europe (SWE), separated by the Strait of Gibraltar [23,24]. A possible explanation, which needs to be tested, is that balancing selection slowed down or prevented genetic differentiation among some populations.

Actually, the case of Gibraltar is particularly puzzling, because no general agreement is found on the role of this strait as a barrier to gene flow. Such a barrier appears to be significant according to classical markers [25], Alu insertions [26,27], X-chromosome SNPs [28], and, most of all, the Y chromosome [29-33], although traces of gene flow have been detected for the latter [34]. By contrast, studies on mtDNA [35-37], nuclear and Alu STRs [38,39] and the GM polymorphism [24,38,40,41] indicate that the strait was permeable to human migrations, with an estimated contribution of North-West Africa to Iberia of 18% for mtDNA [36], compared to 7% for Y-chromosome lineages [30].

In view of these contradictory results, we focused our study on the genetic diversity among populations located in the Gibraltar region. First, to determine the impact of the Strait of Gibraltar as a barrier to gene flow at distinct prehistoric periods and explain possible differences observed between some genetic polymorphisms; second, to determine whether balancing selection could have resulted in a reduced level of inter-population differentiation at the HLA-DRB1 locus, and, in such case, with what intensity of selection. Considering that the Strait of Gibraltar is a geographic barrier between South-Western Europe and North-Western Africa, one would expect to observe higher inter-population diversity across the Strait as compared to what is observed on both sides of the Strait. Therefore, we used a computer simulation approach within the Approximate Bayesian Computation (ABC) framework [42] to estimate several parameters of population differentiation under alternative scenarios for the peopling history of the West Mediterranean region, and to estimate a selection coefficient for HLA-DRB1.

## Results

### Observed data

Intra- and inter-population indices (see Table 1) indicate that HLA-DRB1 and MNSs are the loci which show the less overall differentiation between populations (*F_ST _*≤ 0.11). It is particularly intriguing that the level of differentiation across the Strait of Gibraltar is not enhanced compared to the level of differentiation within each continental area (SWE and NWA) for these two loci (*F_CT _*<*F_SC _*and *D_inter _*≈ *D_intra_*). ABO shows a low level of overall differentiation (*F_ST _*= 0.017) but a higher level of differentiation across the Strait than within each continental area (*F_CT _*>*F_SC _*and *D_inter _*>*D_intra_*). MtDNA shows a higher level of overall differentiation (*F_ST _*= 0.044) but only a weak increase of genetic differentiation between NWA and SWE compared to genetic differentiation within each region (*F_CT _*= *F_SC _*= 0.22 and *D_inter _*>*D_intra_*). RH, GM, and especially the Y chromosome show a much higher level of overall differentiation between populations and a clear distinction between populations from the two sides of the Strait (*F_CT _*>*F_SC _*and *D_inter _*>*D_intra_*).

**Table 1:** Diversity indices computed for each of the seven genetic loci analyzed.

		*FSC*	*FCT*	*FST*	*P (FSC)*	*P (FCT)*	*P (FST)*	*D_intra_*	*D_inter_*	*P (D_intra_)*	*P (D_inter_)*		
**ABO**	*Allele Freq*	0.007 (0.002)	0.010 (0.010)	0.017 (0.012)	0.331 (0.000)	0.437 (0.000)	0.697 (0.000)	0.007 (0.005)	0.017 (0.017)	0.061 (0.329)	0.128 (0.660)	3.00 ± 0.02 (3.00)	0.475 ± 0.015 (0.48)
**MNSs**	*Allele Freq*	0.006 (0.005)	0.004 (0.004)	0.010 (0.009)	0.401 (0.000)	0.231 (0.001)	0.983 (0.000)	0.011 (0.008)	0.011 (0.011)	0.079 (0.205)	0.156 (0.346)	4.00 ± 0.01 (4.00)	0.696 ± 0.005 (0.690)
**RH**	*Allele Freq*	0.022 (0.011)	0.047 (0.042)	0.068 (0.052)	0.986 (0.000)	0.997 (0.000)	1.000 (0.000)	0.021 (0.014)	0.066 (0.072)	0.226 (0.473)	0.709 (0.945)	4.79 ± 0.19 (5.56)	0.664 ± 0.009 (0.657)
**GM**	*Allele Freq*	0.012 (0.010)	0.039 (0.040)	0.051 (0.049)	0.814 (0.000)	0.989 (0.000)	1.000 (0.000)	0.014 (0.011)	0.051 (0.053)	0.103 (0.292)	0.522 (0.871)	3.97 ± 0.18 (4.97)	0.54 ± 0.019 (0.538)
**HLA-DBR1**	*Allele Freq*	0.009 (0.008)	0.002 (0.003)	0.011 (0.010)	0.983 (0.000)	0.135 (0.000)	0.997 (0.000)	0.010 (0.009)	0.011 (0.011)	0.239 (0.396)	0.253 (0.567)	9.79 ± 0.26 (11.86)	0.872 ± < 0.004 (0.872)
**Y-STR**	*STRs*	0.085 (0.031)	0.222 (0.192)	0.288 (0.217)	1.000 (0.000)	1.000 (0.000)	1.000 (0.000)	0.110 (0.084)	0.359 (0.299)	0.330 (0.468)	0.963 (1.00)	11.83 ± 0.60 (42.59)	*0.863 ± 0.031 *(0.874)
**Y-SNP**	*SNPs*	0.0511 (0.049)	0.470 (0.446)	0.497 (0.473)	0.734 (0.000)	1.000 (0.000)	1.000 (0.000)	0.065 (0.054)	0.723 (0.710)	0.425 (0.308)	0.681 (1.00)	5.334 ± 1.60 (8.30)	*0.592 ± 0.193 *(0.574)
**mt-HVS1**	*DNA seq*	0.022 (0.027)	0.022 (0.020)	0.044 (0.047)	0.988 (0.000)	0.963 (0.000)	1.000 (0.000)	0.021 (0.025)	0.043 (0.045)	0.203 (0.386)	0.553 (0.844)	19.03 ± 4.45 (32.96)	*0.92 ± 0.070 *(0.938)

The first line represents the mean value for 10,000 resamplings while the second line (in brackets) represents the statistics computed using the whole dataset. *P(x) *represents the proportion of indices statistically significant at 1% level or the exact *P *value for the whole dataset. *D *stands for Reynolds genetic distances,  for the average number of alleles over the samples and  for the average heterozygosity (*gene diversity) over the samples

**Figure 1:**
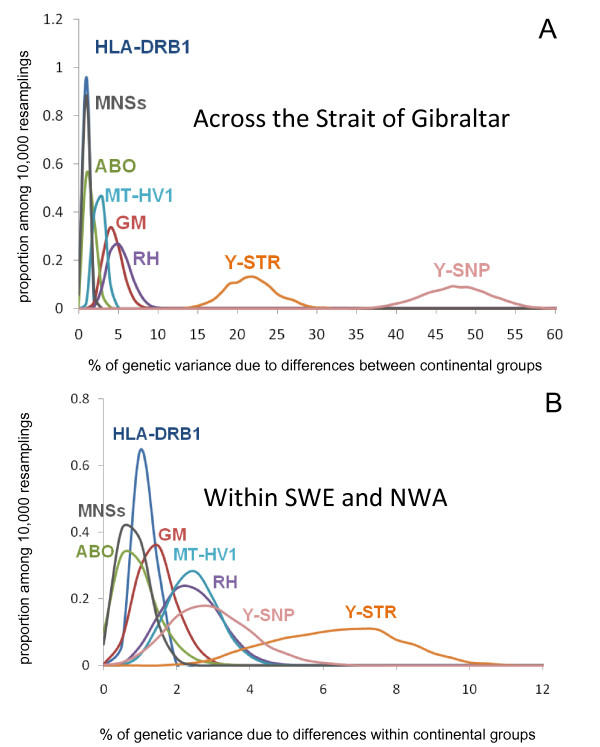
**Proportion of genetic variance (average value) due to differences between the two continental groups of populations South-Western Europe (SWE) and North-Western Africa (NWA) (A) and within both groups (B)**.

Figure 1 displays the percentages of genetic variance due to differences between populations, between the two continental groups (SWE and NWA) and within both groups. It confirms graphically the observations described above. ABO, and especially MNSs show a low overall genetic differentiation between populations (both within and between continental groups), while HLA-DRB1 shows a strong reduction of differentiation across the Strait of Gibraltar, despite the high level of polymorphism at this locus. At the opposite, a very strong "genetic barrier" across the Strait is confirmed for the Y chromosome [30], that is even more pronounced with SNP data than with STRs. GM, RH and mtDNA show intermediate levels between those of HLA-DRB1 and the Y chromosome.

As potential pathogen receptors of the red blood cell membrane, ABO and MNSs blood groups may be affected by selection [8,10,43-45]. Although the Ewens-Watterson and Slatkin's exact tests of selective neutrality did not show any significant deviation for ABO after Bonferroni's correction (559 samples tested, Additional file 1: Supplemental Table S1), neutrality tests performed on ABO typed at the molecular level are clearly significant [8]. For MNSs and HLA-DRB1, a majority of populations (about 60% and 100%, respectively) exhibit a significant excess of heterozygotes through Ewens-Watterson and Slatkin tests, although selective neutrality is only rejected for HLA-DRB1 after Bonferroni's correction for multiple tests. RH and GM appear to be selectively (nearly) neutral, as rejections of Ewens-Watterson and Slatkin's exact tests are a minority (0% and 27% for GM and RH, respectively) and no rejection is observed after correction for multiple tests. Consequently, we decided to perform all further demographic estimations (see below) by using only RH and GM (in addition to mtDNA and the Y-chromosome).

### ABC estimation

We performed simulations using the two programs SELECTOR and SPLATCHE [46] combined to the ABC approach [42] in order to: 1st estimate the impact of the Strait of Gibraltar on population migration (using the four loci presumably evolving in a (nearly) neutral way: GM, RH, mtDNA and the Y chromosome); 2nd estimate the selection coefficient required to produce the reduced genetic differentiation observed at the HLA-DRB1 locus (and, additionaly, at the MNSs and ABO loci).

### Evaluation of the scenarios

The first goal was to evaluate which scenario(s) among the 4 proposed is (are) the most compatible with the observed data. In a few words, we simulated: 1) a "Palaeolithic" scenario *P *with gene flow between small-sized populations since pre-Neolithic times (starting ~20,000 years before present (BP); 2) a "Neolithic" scenario *N *with gene flow between large-sized populations since the Neolithic transition (starting ~10,000 years BP); 3) a scenario *PN *combining the first two ones; 4) a scenario *PNI *which also considers the expansion of the Arabian empire and diffusion of Islam into Maghreb. See the Material and Method section for more details about those scenarios. As ABC estimation needs many replicates and thus a huge computer power, we decided to perform the estimation of parameters only under the best scenario(s). We skipped ABO, MNSs and HLA-DRB1 at this stage, because we wanted to study the impact of balancing selection on those loci at a later stage. We ran 100,000 simulations for each of the 4 scenarios (*P*, *N*, *PN*, *PNI*). Then, using the ABC approach, we retained the 0.25% best simulations among the 400,000 simulated and we looked at the proportion of those simulations which belonged to any of the 4 alternative scenarios.

**Figure 2:**
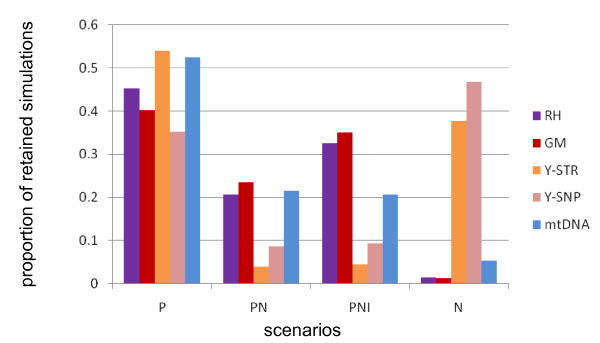
**Proportion of the best 0.25% simulations among 400,000 for each of 4 alternative scenarios and RH, GM, Y-chromosome and mtDNA loci**. P stands for Palaeolithic scenario, N for Neolithic, PN for Palaeolithic and Neolithic and PNI for Palaeolithic, Neolithic and Islamic expansion (see text for details).

As shown in Figure 2, in all cases (but Y-chromosome SNPs) the most probable scenario is scenario *P*. At the opposite, scenario *N *is incompatible with the data (below 5%) except for the Y chromosome. Scenarios *PN *and *PNI*, including both the Neolithic transition and the Arabian conquests, do not fit the data better than scenario *P *alone. Consequently, we decided to perform all further estimations under scenario *P*, which best explains the data. In order to evaluate the effect of deme size on the results, we also performed an identical scenario evaluation by using a smaller grid (64 demes of about 100 x100 kilometers) with a much reduced number of simulations (80,000 overall). Supplemental Figure S10 (Additional file 1) shows that the results are globally robust to deme size, with the notable exception that scenario *N *is favoured over scenario *P *for the Y-chromosome STRs.

### Estimation of parameters

**Figure 3:**
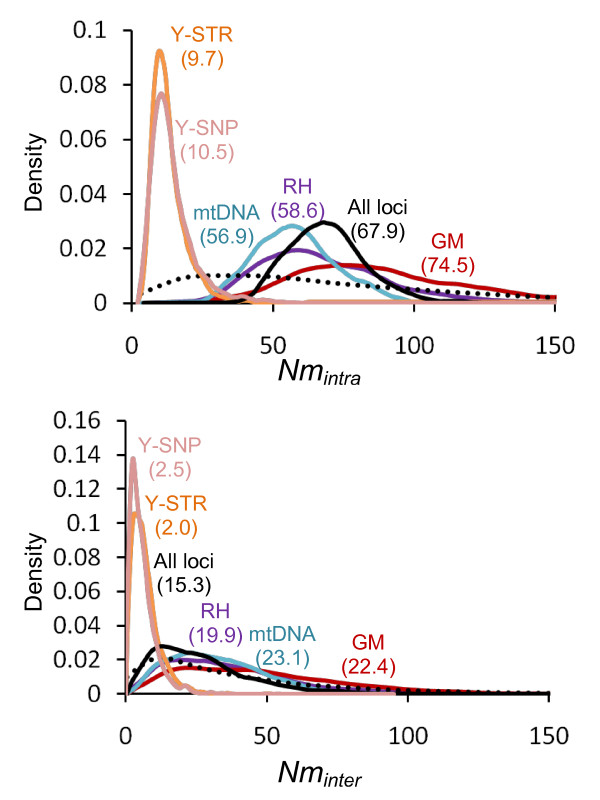
**Curves representing the prior (dotted line) and posterior distributions (plain lines) obtained for the *Nm_intra _*and *Nm_inter _*parameters of scenario P and 4 genetic loci: RH, GM, mtDNA and Y chromosome ("Y-STR" and "Y-SNP") as well as the estimation for the 4 loci taken together ("All loci")**. The mode of the distribution is given in brackets.

Still using the ABC approach, we estimated the composite demographic parameters *Nm_intra _*and *Nm_inter _*for RH, GM, mtDNA and the Y chromosome (Figure 3). We decided to estimate composite parameters rather than single parameters *K*, *m_intra _*and *m_inter _*because the proportion of parameter variance explained by the summary statistics (estimated by the coefficient of determination *R^2^*) is substantially higher for *Nm_intra _*and *Nm_inter _*(see Additional file 1: Supplemental Table S4), which indicates that composite parameters have a higher potential to be correctly estimated. At the opposite, the growth rate *r *has a very low potential to be correctly estimated (*R^2 ^*< 10%). We thus only present and discuss the estimations for *Nm_intra _*and *Nm_inter _*below.

**Figure 4:**
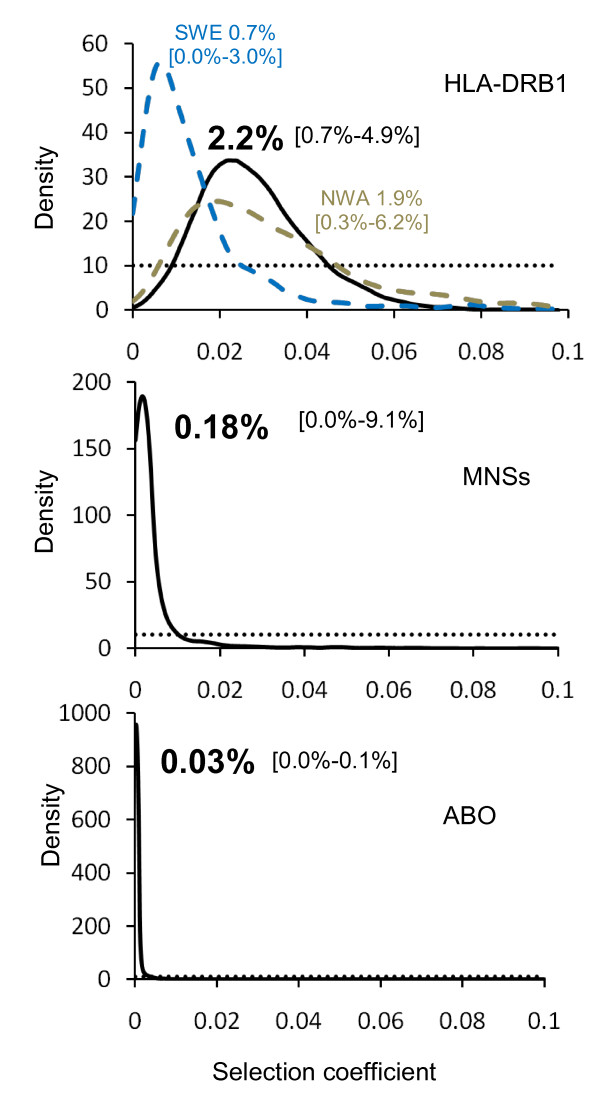
**Curves representing the prior (dotted black line) and posterior distributions (plain line) obtained for the selection coefficient *s *with scenarios P at HLA-DRB1 as well as MNSs and ABO loci**. The modes and the 90% CI of the posterior distributions are given. For HLA-DRB1, *s *has also been estimated independently in North-Western Africa (in dashed grey) and South-Western Europe (in dashed blue), see text for details.

For ABO, MNSs and HLA-DRB1, we also estimated the selection coefficient *s*. These estimations have been performed under scenario *P*. Results are presented in Figure 4 and Supplemental Table S3 (Additional file 1). We took the mode of the weighted posterior distribution as the point estimator because performance tests show that this statistic performs generally better than the mode and the mean, especially for the coefficient of selection (not shown).

#### Performance evaluation

The performance evaluation shows that *Nm_intra _*is the parameter which has the best potential (Additional file 1: Supplemental Table S4) and which is by far the better estimated (maximum bias equal to 0.21, see Additional file 1: Supplemental Tables S5 to S9). This parameter is particularly well estimated for allele frequency data and the multi-locus estimation, while it is slightly underestimated for the two haploid loci. Overall, the performance test shows that the true value is found within the 95% CI in at least 95% of the cases. We can thus be confident in the estimation of *Nm_intra_*. The point estimate for the coefficient of selection *s *may be relatively imprecise (tendency to a 25-50% overestimation, Additional file 1: Supplemental Table S5). However, the 95% CI shows a good coverage (true value found within this interval in more than 93% of the cases) and we can thus consider this CI to be reliable. Note that *s *is better estimated when *Nm_inter _*is low, suggesting that estimating *s *should be performed in regions where a barrier to gene flow exists. The growth rate *r *has a low potential to be correctly estimated (8% as a maximum coefficient of determination *R^2^*). The lack of information about this parameter in our dataset is confirmed by the 95% CI of the posterior distribution which encompasses a very large portion of the prior (Additional file 1: Supplemental Table S2). Unfortunately, one of our main parameters of interest, *Nm_inter_*, is very poorly estimated despite a good potential (coefficient of determination *R^2 ^*~ 0.5, Additional file 1: Supplemental Table S4). Only the 95% CI shows a relatively good coverage (> 77%) and we consequently focus on this CI when interpreting the results. We believe that the intra-continental statistics that we used to measure the reduction of gene flow across the Strait of Gibraltar (*D_inter _*and *F_CT_*) are not sensitive enough (and/or not numerous enough) to estimate *Nm_inter _*with precision. In other words, we do not have sufficient information about this particular parameter in our dataset. Moreover, our representation of the Strait of Gibraltar as a simple barrier to gene flow may be too simplistic to capture accurately its complex demographic role (see Discussion).

#### Estimation

##### Nm_intra_

If we look at the 95% CI for the multi-locus estimation ("All loci"), we get values between 43.6 and 97, with a mode of 67.9 (Figure 3). Locus-independent estimations give point estimates at 74.5 for GM (95% CI between 34.4 and 154), 58.6 for RH (95% CI between 28.1 and 115.6), 56.9 for mtDNA (95% CI between 31.3 and 86.5) and 9.7/10.5 for the Y chromosome STRs/SNPs (95% CI between 4.0 and 25.0 and between 2.9 and 29.5 respectively). Adding sex-specific *Nm_intra _*estimated for females (~57) and males (~10) gives a number (~67) close to the estimations obtained for nuclear loci (~75 and ~59), as theoretically expected. *Nm_intra _*estimated for the Y-chromosome is thus more than 5-fold lower compared to that for mtDNA.

##### Nm_inter_

Despite the fact that the information about *Nm_inter _*that can be drawn from our dataset is relatively limited (see above), we get the following 95% CI for this parameter: 4.2 to 64.7. The point estimate is 15.3, thus about 4 times smaller than the estimation for *Nm_intra_*, but this direct comparison has to be taken with caution given the large CI around *Nm_inter _*(see also the Discussion).

##### r

It seems that there is not a lot of information about this parameter, as the estimated interval encompasses almost all the prior distribution: 0.05 to 0.44 (prior uniform between 0.05 and 0.5). The point estimate is 0.9 but it varies largely over the loci (minimum 0.08 for GM and maximum 0.42 for mtDNA). This estimation is thus certainly not very reliable.

### Estimation of the selection coefficient for HLA-DRB1

Figure 4 (see also Additional file 1: Supplemental Table S3) shows that we obtain a mode of the posterior distribution of the coefficient of selection *s *at 2.2% (see Discussion for *s *estimates obtained independently for South-Western Europe (SWE) and North-Western Africa (NWA) as well as for ABO and MNSs). The performance evaluation shows that this value may be overestimated by 25%-30% (Additional file 1: Supplemental Table S5), suggesting a corrected value close to 1.5%. The 50% CI is between 1.6% and 3.2%, while the 95% CI is between 0.7% and 5.5%. A similar estimation done on a grid made up by 64 demes of 100 × 100 km gives an estimation of 1.8% for *s *with a 95% CI between 0.4% and 4.1% (see Additional file 1: Supplemental Figure S12).

## Discussion

### Loci comparison

The Mediterranean area is a key region for the study of human genetic differentiations as it represents a natural geographic boundary between Europe and Africa, with people of different cultural backgrounds located on both sides. From a genetic point of view, North Africans are closer to Southern Europeans than to sub-Saharan Africans [47], but different markers reveal heterogeneous results regarding the amount of genetic differentiation or gene flow between the northern and southern shores of the Mediterranean Sea [25-35,37,38,40]. In this study, we focused on the region of the Strait of Gibraltar encompassing the Iberian Peninsula and Western Maghreb to try to understand the genetic patterns exhibited by different classical and molecular loci in relation both to natural selection - in particular that affecting the HLA locus - and demographic history. To this aim, we chose 7 independent loci (ABO, RH, GM, MNSs, HLA-DRB1, mtDNA and the Y chromosome) evolving under different evolutionary forces and all tested in a representative number of populations located on both sides of the Strait.

After applying an appropriate re-sampling procedure on the observed data to overcome the problem of data heterogeneity among the different loci, we first estimated several statistics describing genetic diversity in the region under study (Table 1 and Figure 1). Three loci - ABO, MNSs, and HLA-DRB1- showed a very low level of differentiation among populations, with (almost) no difference across Gibraltar compared to what was observed on both sides of the Strait. The first two loci code for blood group antigens usually typed by serological methods. ABO exhibits very homogeneous frequencies of its classical A, B and O alleles at the worldwide scale, except in Amerindians where O is almost fixed in most populations [47]. The global distribution of MNSs haplotype frequencies is more heterogeneous, but, similar to ABO, the patterns are not easily interpreted in relation to the geographic distribution of human populations. Like for many other blood groups, the molecular basis of ABO and MNSs have been clarified recently and previous suspicions of natural selection acting on these systems [48] have been confirmed by population genetics analyses: actually, ABO is one of the most polymorphic genes in humans [8]. Although neutrality tests performed on classical ABO frequencies do not allow to conclude to any kind of selection (this study), this polymorphism shows clear evidence of balancing selection at the molecular level, in particular within the O null-allele class [8,10]. Interestingly, only 0.02% of the genetic variance is accounted for by differences among populations within the O alleles [8], which is less than 7-9% estimated for the HLA-B, -C and -DRB1 loci [49] and much less than the 15% average estimated for other classical and DNA polymorphisms [50-52]. Balancing selection is thus also compatible with the observed apportionment of ABO genetic diversity.

The MN polymorphism is determined by the glycophorin A (GYPA) gene for which significant departures from neutral expectations towards an excess of heterozygotes have been confirmed at the population level both on allele frequencies (Additional file 1: Supplemental Table S1) and on molecular data [43] (on the other hand, the Ss polymorphism defining GYPB does not show almost any deviation from neutrality according to our tests). These results argue in favour of the "decoy hypothesis" whereby GYPA receptors, the most abundant on the erythrocyte surface, would attract pathogens and prevent them to affect more vital tissues [43]. However, the rapid evolution of human glycophorins may have also been driven by *P. falciparum*, ("evasion hypothesis") as both GYPA and GYPB are receptors of this malaria parasite [44]. In the present study, the low proportion of genetic variance due to differences between North-Western Africa and South-Western Europe is almost as extreme for MNSs than for HLA-DRB1 (Table 1 and Figure 1). However, the estimated selection coefficient is much higher for HLA-DRB1 (*s *= 2.2%, CI 95% [0.7.-5.5%]) than for MNSs (*s *= 0.2%, CI 95% [0.0-9.1%]), and is not significantly different from zero for the latter (Figure 4). Also, we found *s *= 0.0, CI 95% [0.0-0.3%] for ABO. Therefore, our study suggests that natural selection had a significant influence on the evolution of HLA-DRB1 but is not - or no more - detectable on the other two loci. Other studies failed to demonstrate the consequence of balancing selection on HLA genetic patterns despite clear evidence of deviation from neutrality [1]. This is probably because natural selection is weak on this gene (e.g. *s *= 2.2% for HLA-DRB1 compared to 10-20% for G6PD/A- [53] and 4-9% for HbC [54], two cases of selection linked to malaria) and would only be detectable by exploring regions where gene flow is reduced (like across geographic barriers) and where differences with neutral markers would be unambiguous. In addition, natural selection may have operated at unequal intensities in different environments (e.g. in regions characterized by different levels of pathogen richness or prevalence of specific diseases), leaving heterogeneous signals in the genetic pool of human populations. We tested this latter hypothesis on our HLA-DRB1 data by estimating *s *independently in NWA and SWE. Interestingly, we found that *s *was higher in NWA (1.9%, CI 95% [0.3%-6.2%]) than in SWE (0.7%, CI 95% [0.0%-3-0%]) where it is not significantly different from zero (Figure 4). These results suggest that the two regions may have undergone a different environmental history, which is a reasonable hypothesis over the 20,000 years period chosen for our simulations, during which important climatic variation occurred (the beginning of this period corresponds to the last glacial maximum, or LGM).This opens new perspectives for the study of human genetic history where the genetic patterns of partially selected polymorphisms like HLA would be explored in relation to environmental factors varying in space and time, in addition to other parameters.

In sharp contrast with ABO, MNSs and HLA-DRB1, the level of genetic differentiation among populations appears to be particularly high across the Strait of Gibraltar for the Y chromosome. Y-chromosome markers are known to discriminate populations and groups of populations much more than other polymorphisms, with a global variation among populations of 33-39% [55,56]. Despite the fact that the estimations of gene flow (*Nm*) on each side of the Strait of Gibraltar or across it are remarkably similar for STR and SNP datasets, the genetic differentiation (*F_CT_*) between NWA and SWE measured with SNPs is more than twice that measured with STRs. This result, which was reproduced in an analysis of a smaller dataset that included exactly the same individuals tested for SNPs and STRs (26 samples, total *n *= 1552 Y chromosomes, *F_CT _*of 44.2% and 26.6% for SNPs and STRs, respectively), is independent of the pattern of differentiation among populations: population pairwise *F_ST_s *(*R_ST_s *for STRs) were indeed highly correlated (*r*= 0.987). Genetic differentiation measured by STRs could be lowered because of the specific mutation process driving the evolution of microsatellite loci, which can produce alleles identical in state but not identical by descent, thereby rubbing out the effect of genetic drift [57-60]. However, Rousset [61] has shown that homoplasy has no simple effect on *F_ST_*, because this measure is not only affected by the mutation rate at microsatellite loci but also by the mutational model governing them. On another hand, a recent study that compared large-scale SNP and STR genotyping in the Human Genome Diversity Panel (HGDP) concluded that SNP-based *F_ST_s *could be inflated by ascertainment bias [62]. It seems thus plausible that a combination of factors, i.e. ascertainment bias in Y-chromosome SNPs and homoplasic effects in Y-chromosome STRs concur here to make estimations of population subdivision diverge. Note also that we encountered problems to reproduce by simulation some characteristics of both Y-chromosome SNP and STR datasets: *i.e*. the very high variance of genetic diversity between samples for STRs (see the standard deviation for the gene diversity *sd H *in Additional file 1: Supplemental Figure S8) and the very high genetic differentiation between continents for SNPs (see *D_inter _*in Additional file 1: Supplemental Figure S9). This discrepancy between observed and simulated statistics could be due either to an overrepresentation of frequent mutations in the SNP dataset [63] or to a choice of very polymorphic STRs (i.e. for forensic purposes), a kind of ascertainment bias that we are not able to reproduce by simulation.

Whichever the nature of the markers used, the remarkable finding of higher levels of continental subdivision associated with the Y chromosome than with other polymorphisms could be due to the fact that haploid components of the genome are more influenced by genetic drift and selection than diploid genes, due to their smaller effective population size [64]. However, a very different pattern (i.e. only a weak genetic barrier at the Strait of Gibraltar) is observed in this study for mtDNA, which is also haploid, thus arguing for a higher female effective population size [56]. The peculiar behaviour of the Y chromosome could then indicate some sex-specific history of migration in the Mediterranean area, with a major demographic effect of males in both Europe and North Africa, at least during the Neolithic [65], [66], and significant female gene flow across the Strait of Gibraltar. Therefore, although contradictory results have been obtained elsewhere between observed mtDNA/Y-chromosome diversity patterns and their expectations based on patrilocality and matrilocality [56,67], a higher level of female migration, as that observed at a global scale [68], is here evidenced for the first time across a sea barrier. Finally, beside sex differences in migration rates, another possible explanatory factor for higher female than male effective population size that is receiving more attention now is a higher variance in reproductive success for males than for females [69,70]. All the hypotheses given above to explain the results of the Y chromosome are of course not mutually exclusive.

RH, GM and mtDNA exhibit close and intermediate proportions of genetic variation across the Strait of Gibraltar, compared to ABO, MNSs, and HLA-DRB1, on one side, and the Y chromosome, on the other side. We thus consider that they are closer to an average for neutral markers, with a significant *F_CT _*between 2.2 and 4.7% across the Strait, and a genetic variation (*F_SC_*) of 1.2 to 2.2% on both sides of the Strait (Table 1 and Figure 1). This result is particularly relevant because close values are found for two nuclear loci (RH and GM, described by frequency data) and one sex-specific molecular marker (mtDNA, described by DNA sequences), which are *a priori *difficult to compare.

### Ancient genetic pattern

Because demography is acting simultaneously on the whole genome (contrary to selection which acts locally), we used 4 loci (RH, GM, mtDNA and the Y chromosome) to infer the demographic scenario which best fits the current genetic structure around the Strait of Gibraltar (Western Mediterranean). Our simulations show without ambiguity that the genetic pattern observed in the Western Mediterranean was mostly constituted in pre-Neolithic times. Indeed, the most probable *scenario *(*P*) involves gene flow since 20,000 years, not only between populations located on both sides of the Strait but also across the Strait. Time elapsed since the Neolithic transition was too short to allow for the current genetic structure to emerge during this period. This is revealed by the very low probability associated to *scenario N *compared to all other *scenarios *involving gene flow during the Palaeolithic (Figure 2). This result is compatible with the fact that the genetic pool of South-Western Europe (in particular the Iberian Peninsula) has been only weakly modified by the Neolithic transition [71] and that the genetic impact of the Neolithic transition in North Africa has been limited to eastern regions according to classical genetic markers [25], although the picture is less clear for mtDNA [72]. The notable exception is the Y chromosome for which a non-negligible proportion of simulations starting in the Neolithic period give compatible results. It has been suggested that the Y-chromosome genetic structure observed in both North Africa [65] and Europe [66] is mainly the result of early food-producing societies, which matches rather well our observations. However, we cannot be conclusive about the scenario that best fits Y-chromosome diversity because scenarios *N *or *P *may be alternatively preferred depending on marker types (SNPs and STRs, Figure 2) and deme size (Additional file 1: Supplemental Figure S10). Moreover, as already stated above, our simulations of Y-chromosome data failed to reproduce the actual data with as much accuracy as they did for the other genetic systems.

It is relatively surprising that we do not obtain a better fit to the observed data when considering the Neolithic transition and the Arabian conquests, in addition to the gene flow occurring in the Palaeolithic era (Scenarios *PN *and *PNI*, Figure 2). The first obvious explanation is that our simple models for the Neolithic transition and Arabian conquests do not capture the principal features of those two events. Alternatively, recent demographic events would not have substantially disturbed the genetic pattern established during the Palaeolithic, which seems compatible with recent theoretical studies suggesting a strong inertia of local genetic pools [73]. In any case, our results support the view that the genetic impact of the Arabian conquest in the Maghreb has been limited, particularly in Morocco and even less in the Iberian Peninsula which was invaded mostly by Maghreb Berbers under Arab leadership [30]. More refined modelling would be necessary to better study the impact of those events on the genetic structure.

### Gene flow on both sides of the Strait of Gibraltar

Our results show that gene flow between populations either within South-Western Europe or within North-Western Africa is not particularly reduced. We compensated the relative lack of precision of the point estimates obtained individually with each marker by multi-locus analyses. *Nm_intra _*is thus estimated between 43.6 and 97 in our study. This estimation is lower than the estimation of 164 +/- 21 obtained for a worldwide STR dataset [74] but is concordant with another estimate obtained for post-Neolithic populations from mtDNA (> 20 [75]). Under our model, *Nm_intra _*represents a rough estimate of the mean gene flow between populations in the studied area since the Last Glacial Maximum (~20,000 years ago). This rough estimate neither takes into account the variation of *Nm *over time, nor at specific periods such as after the Neolithic transition.

Unfortunately, we did not obtain very precise estimations for the other demographic parameters, notably *Nm_inter _*which measures gene flow across the Strait of Gibraltar. We estimated a *Nm_inter _*between 4.2 and 64.7 with respect to the mean gene flow between populations located on each side of the Strait (within South-Western Europe and within North-Western Africa) but the overall reduction is not as strong as the one estimated for the Y chromosome (*Nm_inter _*= 2). This rough estimation confirms that the Strait of Gibraltar does not constitute such a strong barrier as suggested by Y-chromosome data. For comparison, *Nm *estimated on the basis of mtDNA for nowadays hunter-gatherer populations is smaller than 5 [75]. Substantial gene flow across the Strait is not particularly surprising considering that its width has been at maximum equal to 12 kilometres (present time). Our main explanation of the wide interval obtained for the estimation of *Nm_inter _*is that our model lacks certain features that may have impacted on the level of gene flow between populations across the Strait of Gibraltar: *i*) migrations may have been periodical rather than continuous over time. One can imagine that gene flow across the Strait resulted from the movement of groups of individuals at several periods of time, due for example to climatic changes (sea-level up and down) or for cultural reasons; *ii*) the Mediterranean Sea had a profound impact on exchanges between populations located around it [24,76], but its exact role as a vector or barrier to migration may have been variable in time and in different regions. In particular, the Mediterranean Sea may have promoted east-west migration along its coasts but its influence on north-south migration is uncertain. Our model of constant gene flow is maybe too simplistic to capture the impact of maritime movements over the Mediterranean Sea; *iii) *very different migration patterns for males and females across the Strait may also contribute to blur the signal.

### Balancing selection at HLA-DRB1

We obtained an estimation of about 2% for the coefficient of selection independently of the deme size considered (Figure 4 and Additional file 1: Supplemental Figure S12). This coefficient is very close to that estimated at HLA-DRB1 by Satta et al. [16] from the comparison of pairwise differences in silent and replacement sites in the peptide binding region of the molecule (*s *= 1.9% under their model II). Such similarity indicates that this result is very robust, although Slatkin and Muirhead [77] found a lower value based on a simpler model. The close agreement between the molecular studies of Satta *et al. *and ours, despite completely different approaches, indicates that our method is powerful and that a similar approach is likely to be applied successfully to estimate coefficients of selection at the other HLA loci as well. The very good fit of the two estimations obtained in the present study with the two different grid size also suggests that our estimation is relatively robust although our performance tests demonstrate a tendency to an overestimation of the coefficient of selection (between 25% and 30%, Additional file 1: Supplemental Table S5). One interesting result is that the precision of the estimation is inversely proportional to *Nm_inter_*, the largest bias being obtained when gene flow between North-Western Africa and South-Western Europe is the highest (e.g. bias = 28% with *Nm_inter _*= 8, bias = 50% with *Nm_inter _*= 40, Additional file 1: Supplemental Table S5). Consequently, the estimation of *s *would certainly be improved if we were able to better characterize *Nm_inter _*than in the present study, as both parameters are strongly linked. This indicates that further studies aiming to estimate selection at HLA loci ought to be performed in areas where gene flow is reduced.

## Conclusion

While contrasted conclusions were obtained by previous studies based mostly on single genetic loci, our study clarifies the role of the Strait of Gibraltar regarding its permeability to gene flow. Indeed, our multi-locus approach led us to take into account variations between loci when trying to infer past history of human populations around the Gibraltar area. We were thus able to show that the Y chromosome on one side, and HLA-DRB1 on the other side constitute two extreme cases of very strong and very weak (respectively) genetic differentiations between populations across the Strait. The lack of genetic differentiation for HLA-DRB1 is particularly interesting because it can be explained by balancing selection (with a coefficient of selection estimated here to be around 2%). Given the huge worldwide dataset available for this locus, a better understanding on the mechanisms of selection at HLA loci could be very helpful to the study of human evolution, and more generally MHC. Our results obtained for Gibraltar have to be confirmed by further studies in other areas, especially where gene flow between populations is reduced. This work thus constitutes a step forward towards a better characterization of the combined effects of selection and demography on the genetic structure of populations, and especially on their genetic differentiation.

## Methods

### Data

We compiled data from populations located on each side of the Strait of Gibraltar by gathering published information for various genetic loci from the following countries: Continental Portugal, Spain and France, for South-Western Europe (SWE), and Morocco, Tunisia and the Northern part of Algeria (North of latitude 27.7°), for North-Western Africa (NWA). We restricted our compilation to markers for which many population samples were available for both sides of the Strait of Gibraltar in order to get enough information about the level of gene flow in each side of the Strait as well as across the Strait. Data on 7 different genetic loci were thus collected (Table 2), five diploid (ABO, RH, GM, MNSs and HLA-DRB1) and two haploid (mtDNA and the Y-chromosome). The four classical diploid markers (ABO, RH, GM and MNSs) are represented by samples tested using immunological techniques, whereas HLA-DRB1 and the two haploid loci have been typed at the DNA level (SSOP for HLA-DRB1, DNA sequencing for mtDNA HVS1 and 5 STRs and 16 SNPs from the non-recombining part of the Y chromosome). We only used populations whose sample sizes were equal to or larger than 50 individuals for the diploid loci, and 30 individuals for the haploid loci (19 for the Y-chromosome SNP dataset). ABO data are represented by the frequencies of 3 alleles: A, B and O; RH data by the frequencies of 8 haplotypes: CDE, CDe, CdE, Cde, cDE, cDe, cdE and cde; and MNSs data by the frequencies of 4 haplotypes: MS, Ms, NS and Ns. We also used frequency data for HLA-DRB1 (13 different alleles: DRB1*01, *02, *03, *04, *07, *08, *09, *10, *11, *12, *13, *14, "blank") and for GM (15 different haplotypes: GM*1,17;21, *1,2,17;21, *3;5,10,11,13,14, *1,17;5,10,11,13,14, *1,17;10,11,13,15, *1,17;10,11,13,15,16, *1,3;5,10,11,13,14, *1,17;5,6,10,11,14, *1,17;5,6,11,24, *3;5,10,11,13,14,15,24, *1,3;21, *1,2;17,5,10,11,13,14, *3;-, *-,17;21, *1,17;5,6,10,11,13,14, *-;5,10,11,13,14, *17;5,10,11,13,14, "other"). We used 5 STR loci for the Y chromosome: DYS19, DYS390, DYS391, DYS392 and DYS393. A second set of markers was also used for the Y chromosome, incorporating the following 16 SNPs: M145, M2, M35, M78, M81, M123, M89, M201, M170, 12f2, M172, M9, M45, M173, M17, M124. For mtDNA, we used HVS1 sequences between nucleotide positions 16090 and 16365, excluding positions 16182 to 16193 (data compiled in [78]).

**Table 2:** Number of samples in South-Western Europe (SWE) and North-Western Africa (NWA)

*Locus*	*SWE*	*NWA*	*All*	*n *± *stdev*	*Na*	*Ref*
ABO	451	113	564	7,610 ± 19,983	3	[48,99-101]
MNSS	10	26	37	419 ± 579	4	[25,48,100,102-107]
RH	30	18	48	870 ± 720,360	8	[48,100]
GM	21	17	38	332 ± 167	15	[108]
HLA-DRB1	10	12	22	216 ± 71	13	[108]
Y-STR	14	13	27	104 ± 61	372	[65,109-114]
Y-SNP	15	8	23	64 ± 34	15	[30,65,115,116]
mtDNA - HVS1	11	14	25	59 ± 21	482	[36,78,117-126]

Mean sample sizes (*n*) and standard deviation (*stdev*), number of different alleles (*Na*) and references for the data used in this work (*Ref*).

### Standardization of the number and size of samples

Following Meyer et al [1], we applied a random sampling procedure from populations frequencies in order to remove the bias due to differences in the number and size of samples for the various loci (see Additional file 1: Supplemental Figure S1). For each genetic locus, we randomly drew 16 different population samples, 8 in SWE and 8 in NWA. For each of these 16 samples, we randomly drew 50 different individuals. We repeated this procedure 10,000 times and we ran all analyses on each of those replicates. In this way, the number of samples and sample sizes were identical for all markers. Note, however, that sizes have been limited to 30 individuals for mtDNA and the Y chromosome STRs and to 19 for the Y-chromosome SNPs, due to smaller samples.

### Intra- and inter-population analyses

We compared the genetic diversity for the various genetic loci across the Strait of Gibraltar based on the different types of markers used (allele or haplotype frequencies, STRs, DNA sequences). We computed two standard diversity indices: the mean number of alleles within samples (*na*) and the mean heterozygosity (*H*). For mtDNA and the Y chromosome, *H *was computed as the gene diversity [79].

To evaluate the genetic diversity among populations, we performed two kinds of analyses using the software Arlequin ver 3.1 [79]: *i) *ANOVA or AMOVA [80] where the populations from SWE and those from NWA were considered as two separate groups; *ii) *Reynolds pairwise genetic distances between populations [81]. When necessary, 10,000 repetitions were used to compute the P-values. These analyses were performed on every resampled dataset (i.e. 10,000 times) in order to get empirical distributions for each statistic.

### Simulations

We developed a simulation program called SELECTOR, written in C++ and compiled on Linux. This program allows one to simulate populations of diploid individuals, generation by generation, within a spatially explicit framework. Allele frequencies in the populations, either at neutral or selected loci, may be simulated. The spatial framework as well as the demographic model used have been derived from the software SPLATCHE [46]. This allows a direct comparison between the outputs of both programs when identical demographic parameters are used. However, contrary to SPLATCHE which uses the coalescent, here the genetic diversity is generated in a forward way, so that overdominant selection on multiallelic loci can be simulated. Various evolutionary scenarios allowing for a combined influence of demography and selection on frequency data may thus be simulated using SELECTOR. Note that forward genetic simulations using SELECTOR require much more computational time than backward genetic simulations using SPLATCHE (about tenfold more). SELECTOR was used to simulate the gene frequencies at ABO, MNSs, RH, GM and HLA-DRB1 loci, while SPLATCHE was used to simulate molecular diversity at mtDNA and the Y-chromosome.

#### Demographic model

SELECTOR simulates diploid individuals, generation by generation, within a stepping-stone framework [82]. The population can thus be subdivided into numerous demes which may exchange migrants at each generation. The demographic model is adapted from the model n°1 described by Currat et al [46]. The number of emigrants *M *from a deme is computed as follows: *M_t _= mN_t_*, where *m *is the migration rate, and *N_t _*is the population density of the deme at generation *t. M_t _*is then distributed equally into the neighbouring demes, and the remaining individuals (or fraction of individuals) are added to the emigrant pool of the next generation. The demography is logistically regulated within each deme as follows: , where *Nt *is the density at generation *t*, *r *is the growth rate, and *K *is the carrying capacity (number of individuals in the deme at demographic equilibrium). All parameters *r*, *m *and *K *can be defined independently for each deme. These parameters can also be modified at any time during the process to allow the simulation of a large variety of demographic scenarios. The program SPLATCHE uses the same demographic model but simulates haploid individuals (see [46] for more details).

#### Selection model

Symmetrical overdominant selection [83] is implemented in SELECTOR. This selection model corresponds to a selective advantage for heterozygous over homozygous individuals, all heterozygotes having an identical fitness [51]. At each generation, new genotypes are created using the allele frequencies within the deme at the preceding generation. If the genotype is homozygous, it may be discarded with a probability equal to *s*, where *s *is the selection coefficient. The relative fitness of homozygous is thus 1-*s*, and 1 is the relative fitness for heterozygous. This relative fitness measures the difference of viability between the two genotypes.

#### Scenarios simulated

We simulated 4 different evolutionary scenarios (*P*, *N*, *PN*, *PNI*) that may potentially account for the genetic structure observed at the Strait of Gibraltar.

##### P

The "Palaeolithic" scenario simulates gene flow between small-sized populations across the Strait of Gibraltar since the Palaeolithic era. The duration of this gene flow is 800 generations (~20,000 years), a time depth which corresponds roughly to the upper limit attributed to the Ibero-Maurusian blade industry [84]. It seems indeed that this tradition marks a major break with preceding technologies [85] and is clearly associated to behaviourally modern humans [86]. The densities at equilibrium vary between 0.03 and 0.6 individuals per km^2^, as those values encompass the density estimates for Pleistocene hunter-gatherers [87,88]. The growth rate is taken between 0.05 and 0.5 [47,63].

##### N

The "Neolithic" scenario simulates gene flow between large-sized populations across the Strait of Gibraltar since the Neolithic era. The starting time is 300 generations (~7,500 years) ago, in agreement with the beginning of the Neolithic transition in South-Western Europe [89], although this transition started later in North-Western Africa [47]. At population equilibrium, the densities are 10 times larger than those chosen for scenario *P *[90], i.e. between 0.3 and 6 individuals per km^2^. The growth rate is two times higher than for scenario *P *[91,92].

##### PN

This scenario combines scenarios *P *and *N*. Hence, the "Palaeolithic" scenario is simulated as described above, but 300 generations before present, the carrying capacities are increased by a factor of 10 and the growth rate by a factor of 2.

##### PNI

We add the first *Islamic *colonisation on top of the *PN *scenario. It is simulated by a migration wave starting 55 generations ago (~625 years A.D., i.e. [40]) from the extreme east of NWA, and moving towards the North of the Iberian Peninsula, via the Strait of Gibraltar. The advance of the *Islamic *colonisation is simulated as a 4-times increase of the migration rate, moving at a velocity of one deme per generation. This results in 8 generations for the wave to move across North Africa (~200 years) and the same across the Iberian Peninsula.

**Figure 5:**
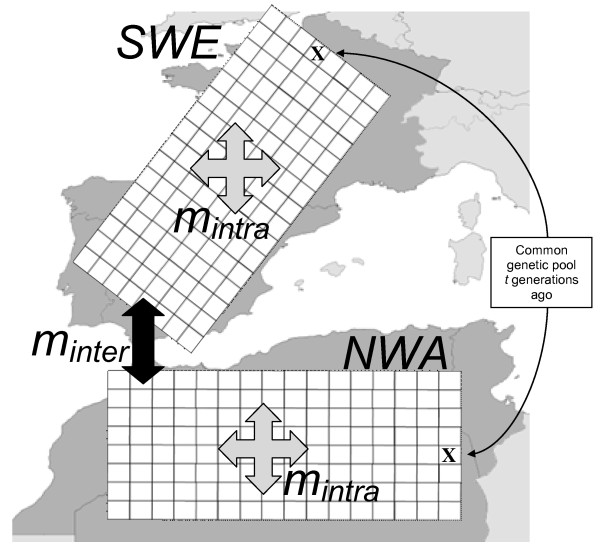
**Schematic representation of the two grids of demes representing South-Western Europe (SWE) and North-Western Africa (NWA)**. The whole simulated area is subdivided into 256 demes. The two crosses represent the demes where the settlement of the areas starts from a common genetic pool *t *generations ago. There are two different migration rates: *m_intra _*is the migration rate between demes within a continent (either SWE or NWA) and *m_inter _*is the migration rate between demes among continents. Continents are connected by 8 demes on each side.

Each scenario was done using a grid made up of 256 demes of about 50 × 50 kilometres, (see Figure 5). The number of migrants exchanged between neighbouring demes at equilibrium is equal to *Km*, but noted *Nm *here for consistency with previous literature. The products *Nm_intra _*and *Nm_inter _*thus measure the mean level of gene flow at equilibrium on each side of the Strait of Gibraltar (intra-continental) and across the Strait (inter-continental), respectively. Note that for autosomal markers, *Nm *corresponds to the effective number of both male and female migrants, while for sex-specific markers, it corresponds either to the number of males (Y chromosome) or to the number of females (mtDNA). For all scenarios, the intra-continental migration rate (within NWA or within SWE, respectively) was chosen between 0.025 and 0.4, which correspond to *Nm *values between 3 and 200 for the "Palaeolithic" scenario. *Nm *is 10 times higher for the Neolithic era, and 40 times higher for the Islamic colonisation. Note that *Nm_inter _*is never higher than *Nm_intra _*in any simulation.

#### Genetic data

To counterbalance the fact that we do not simulate the occurrence of new mutations using SELECTOR, the initial number of alleles of each genetic system is randomly drawn between 3 and 15. This method allows the simulation of multi-allele frequencies whose characteristics at the population level are a good approximation of observed data at ABO, MNSs, GM, RH and HLA-DRB1 loci (see Additional file 1: Supplemental Figures S2 to S6). The alleles drawn are then randomly distributed among two initial populations (marked by "X" in Figure 5), one located in Africa, and the other one in Europe. These two populations then increase demographically and spatially until occupying the whole grid. We thus make the assumption that Europe and Africa were colonised by two populations sharing an ancestral gene pool without specifying the location of this ancestral population.

When simulating molecular data using SPLATCHE, the following parameters are used. We simulate mitochondrial DNA sequences of 265 base pairs length with a mutation rate taken for a uniform prior distribution between 10^-6 ^and 10^-5 ^and a transition rate of 0.0159 [93]. For the Y chromosome, we simulate 5 Short Tandem Repeats (STRs) under the generalized stepwise mutation model (GSM, [94,95]). We use the average estimations taken from the YHRD database for each locus as upper limits for the prior distribution (dys19 = 2.299*10^-3^, dys390 = 2.102*10^-3^, dys391 = 2.599*10^-3^, dys392 = 4.12*10^-4^, dys393 = 1.045*10^-3^[96]). The lower limit is tenfold inferior to the upper limit for each locus. The GSM parameter is randomly drawn in the uniform distribution 0.0 (strict stepwise mutation model) to 0.1. When simulating Y-chromosome SNPs, no mutation rate was used as for each SNP, a mutation is randomly superimposed on the genealogy of the locus, as in [97]. Moreover, the overall minimum frequency for the minor allele was set to 0.1%.

#### Approximate Bayesian Computation (ABC) framework

Our simulation procedure is integrated in the Approximate Bayesian Computation (ABC) framework [42]. This method allows one to evaluate the probability of various evolutionary scenarios as well as to estimate the more probable values for the parameters of the model. We use the ABC approach for two different tasks: 1°) evaluate the relative probability of 4 different plausible simple scenarios (*P*, *N*, *PN*, *PNI*,); 2°) estimate the demographic parameters for the best scenario, and estimate the selection coefficient *s *for HLA-DRB1 (and also for MNSs and ABO).

In accordance with the ABC methodology, we repeat 100,000 times the simulation of a given scenario, each time with input parameters drawn from prior distributions (see Additional file 1: Supplemental Table S2). Euclidian distances are then computed between simulated and observed summary statistics. The basic principle is that simulations that give the smallest distances (e.g. the statistics that are the closest to the real ones) are generated by the most probable combination of parameters and models. As "observed" statistics, we use the mean values obtained after 10,000 resamplings from the original dataset (Table 2). We choose 9 summary statistics for the ABC estimation in order to capture different aspects of the data both at the within-population and at the between-population levels: for all loci, we use the 3 fixation indices *F_CT_*, *F_SC _*and *F_ST _*obtained with AMOVA/SAMOVA analyses [80], the mean pairwise Reynolds distances *D_intra _*between populations within a continental area (NWA or SWE) and *D_inter _*between each pair of populations belonging to different continental areas. For ABO, MNSs, GM, RH and HLA-DRB1, we also use the mean number of alleles (*na*) and heterozygosity (*H*) within samples, as well as their standard deviation. In order to use the molecular information, we use specifically for mtDNA the mean number of segregating sites *S *and the mean molecular diversity π (and their standard deviation) as well as, specifically for the Y-chromosome STRs, the gene diversity *H *and the mean allelic range *R *(and their standard deviation) and the gene diversity *H *and the number of haplotypes *K *for the Y-chromosome SNPs. The following steps are followed for the ABC procedure: 1°) 100,000 simulations are done for every scenario (i.e. 400,000 simulations overall); 2°) Euclidian distances between observed and simulated statistics are computed according to Beaumont et al. [42], using a weighted multiple linear regression. The parameters are transformed following Excoffier et al. [98], using the regression y = log[tan(x)-1], which restricts the posterior distribution of parameters within the range of the prior distribution; 3°) the fraction *f *of the best among the 400,000 simulations is computed. Here we chose *f *= 1,000, which corresponds to 0.25% tolerance level, but our results are robust with various values of *f *(not shown); 4°) for the best scenario, we generate 900,000 additional simulations (one million overall) and we estimate the most probable parameter values from the fraction *f *of retained simulations (again 0.25% tolerance level), independently for each locus; 5°) we also perform a multi-locus analysis by generating 500,000 demographic simulation data for 4 independent markers considered as evolving almost neutrally, i.e. RH, GM, mtDNA and the Y-chromosome STRs. We are conservative when using Y-chromosome STRs instead of SNPs because STRs are probably less affected by ascertainment bias and the dataset is much numerous. We proceed to parameter estimation using jointly the 36 statistics calculated for those 4 loci.

We use SELECTOR and SPLATCHE to perform the simulations and the program abcEst [98] to do the ABC estimation. As the ABC estimation needs a very large number of simulations in order to be efficient, we run our simulations on the "Myrinet" Linux Cluster of the University of Geneva. The accuracy of the estimation for each kind of marker (allele frequencies with or without selection, STRs and DNA sequences) is asset by using a performance test which is described in Additional file 1: Supplemental Table S5 to S9.

## Authors' contributions

MC and ASM conceived and designed the study. MC, ESP and ASM carried out the analyses, interpreted the results and wrote the manuscript. MC developed the simulation program and carried out the simulations and the ABC estimation procedure. All authors read and approved the final manuscript.

## Supplementary Material

Additional file 1 **Complementary information on the methods and results**. This file contains additional information on: 1- Resampling procedure; 2- Ewens-Watterson and Slatkin neutrality tests; 3,4,5,6- ABC estimation procedure, such as prior distributions, detailed estimation results, distributions of statistics and performance evaluation; 7- Methodological details on the simulation of different selection coefficients in Africa and Europe; 8- Results obtained with simulations on a grid with a different resolution. Click here for file
