# Supplementary material for: Human genetic differentiation across the Strait of Gibraltar
Source: BMC Evol Biol. 2010 Aug 3;10:237. doi: 10.1186/1471-2148-10-237 (PMC3020631; doi:10.1186/1471-2148-10-237)
Supplement: Additional file 1 — Complementary information on the methods and results. This file contains additional information on: 1- Resampling procedure; 2- Ewens-Watterson and Slatkin neutrality tests; 3,4,5,6- ABC estimation procedure, such as prior distributions, detailed estimation results, distributions of statistics and performance evaluation; 7- Methodological details on the simulation of different selection coefficients in Africa and Europe; 8- Results obtained with simulations on a grid with a different resolution. [file 1471-2148-10-237-S1.PDF]

Additional file 1

1 – Resampling method to reduce differences in sampling between datasets

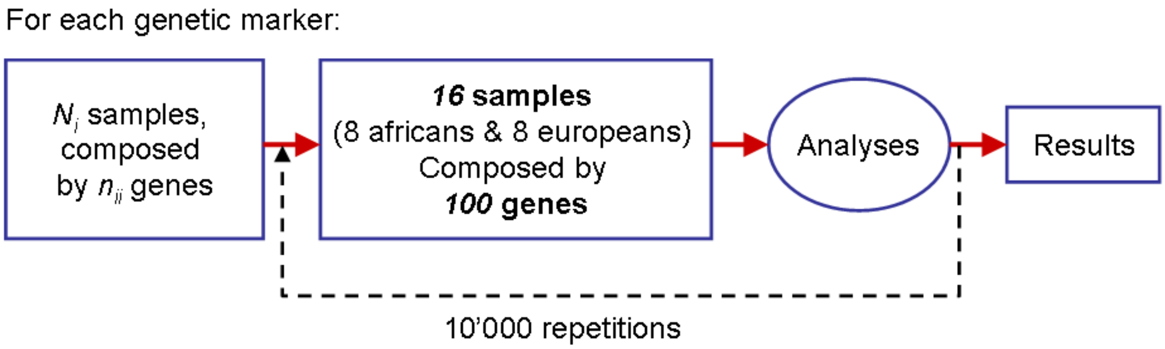

Figure S1 Schematic representation of the resampling strategy used for the data analyses. For each genetic locus, 16 population samples of 50 individuals (30 for the haploid loci and 19 for Y-chromosome SNPs) were randomly sampled 10'000 times from the data and analyzed**.**

2 – Ewens-Watterson and Slatkin's exact tests of selective neutrality

		Ewens-Watterson test				Slatkin's exact test			
Locus	*n*	*P < 0.01*	*P < 0.01**	*P < 0.05*	*P < 0.05**	*P < 0.01*	*P < 0.01**	*P < 0.05*	*P < 0.05**
ABO	559	0	0	4	0	0	0	10	0
MNSs	37	13	0	22	0	12	0	23	0
MN	37	4	0	31	0	4	0	31	0
Ss	37	0	0	1	0	0	0	1	0
RH	45	3	0	12	0	3	0	12	0
GM	38	0	0	0	0	0	0	0	0
HLA-DRB1	22	15	1	22	8	9	0	16	3

Table S1: Selective neutrality tests on ABO, MNSs, MN, Ss, RH, GM and HLA-DRB1 loci. n: number of samples tested (samples with more than 2000 individuals could not be tested due to the limitations of the Arlequin program, and were eliminated for this analysis); P < 0.01: number of tests significant at the 1% level; P < 0.01*: number of tests significant at the 1% level after Bonferroni's correction for multiple tests; P < 0.05: number of tests significant at the 5% level; P < 0.05*: number of tests significant at the 5% level after Bonferroni's correction for multiple tests.

3 – Prior distributions used for the ABC estimation

Parameter	***Prior distributions (all Uniform)***			
	P	***N***	***P****	***N****
Demography				
t (number of generations)	800	300	800	300
k (carrying capacity)	125-500	1250-5000	500-2000	5000-20000
r (growth rate)	0.05-0.5	0.1-1.0	0.05-0.5	0.1-1.0
m_{intra} (migration rate within continent)	0.025-0.4	0.025-0.4	0.025-0.4	0.025-0.3
m_{inter} (migration rate across the Strait)	0.0-0.4°	0.0-0.4°	0.0-0.4°	0.0-0.4°
Genetics				
i (Initial number of alleles for GM, RH, ABO, MNSs and HLA-DRB1)	3-15			
s (balancing selection coefficient for HLA-DRB1, also used for MNSs and ABO)	0.0-0.1			
μ_{mtdna} (mutation rate per generation and per bp for mtDNA)	10^{-6} - 10^{-5}			
μ_{dys19} (mutation rate per generation for *dys19* locus on Y chromosome)	$2.299*10^{-4}$ - $2.299*10^{-3}$			
μ_{dys390} (mutation rate per generation for *dys390* locus on Y chromosome)	$2.102*10^{-4}$ - $2.102*10^{-3}$			
μ_{dys391} (mutation rate per generation for *dys391* locus on Y chromosome)	$2.599*10^{-4}$ - $2.599*10^{-3}$			
μ_{dys392} (mutation rate per generation for *dys392* locus on Y chromosome)	$4.12*10^{-5}$ - $4.12*10^{-4}$			
μ_{dys393} (mutation rate per generation for *dys393* locus on Y chromosome)	$1.045*10^{-4}$ - $1.045*10^{-3}$			
G (GSM parameter for Y chromosome STRs)	0.0 – 0.1			

Table S2 Values of parameters for scenarios *P* (Palaeolithic) and *N* (Neolithic). The number of generations (*t*) is kept constant but all other parameters are drawn from a uniform distribution within the given interval for each simulation. ° m_{inter} is always smaller or equal to m_{intra}. Scenarios PN and PNI are combined versions of P and N (see method section). * values used for the smaller grid used below.

4 – Detailed estimations obtained for each of the 7 loci (ABC estimation)

***Nm* Intra**<br>Prior=Product of two uniform priors (*K* and m_{intra})<br>[3 ; 200]	***Mode***	***Mean***	***Median***	***CI 50%***	***CI 95%***
ABO	112.9	112.5	111.6	86.8-131.9	53.2-177.9
MNSs	125.6	127.4	127.4	109.7-147.5	74.7-180.3
RH	58.6	68.2	64.4	44.6-73.2	28.1-115.6
GM	74.5	88.5	84.1	57.1-95.9	34.4-154.1
mtDNA	56.9	57.8	57.0	45.8 - 65.1	31.3 - 86.5
Y-STR	9.7	13.0	11.5	7.3 - 13.2	4.0 - 25.0
Y-SNP	10.4	14.2	12.3	7.1 – 14.3	2.9 – 29.5
HLA-DRB1(constant selection)	146.5	144.4	145.3	134.1-161.9	104.0-184.2
HLA-DRB1 (different selection in SWE and NWA)	127.6	131.8	131.6	118.8-144.6	93.6-170.8
*Multiloci**	*67.9*	*69.2*	*68.2*	58.5-76.7	*43.6-97.0*
***Nm* Inter**<br>Prior=Product of two uniform priors (*K* and m_{inter})<br>[0 ; 200]	***Mode***	***Mean***	***Median***	***CI 50%***	***CI 95%***
ABO	40.4	61.8	57.5	22.2-75.1	4.0-126.6
MNSs	65.1	71.9	69.1	34.1-87.5	9.1-136.9
RH	19.9	36.5	32.4	11.0-37.5	2.6-80.2
GM	22.4	48.1	43.2	14.6-50.0	4.0-102.9
mtDNA	23.1	32.0	29.9	14.1 - 36.8	4.2 -62.9
Y-STR	2.0	7.1	5.9	1.8 - 6.8	0.6 - 16.9
Y-SNP	2.5	6.0	4.8	1.2 – 5.4	0.3 – 15.0
HLA-DRB1(constant selection)	25.5	58.4	51.9	14.9-57.9	5.9-125.9
HLA-DRB1 (different selection in SWE and NWA)	95.0	78.3	79.2	60.4-109.6	14.9-135.5
*Multiloci **	*15.3*	*33.1*	*31.0*	11.2-35.5	*4.2-64.7*
**Growth rate *r***<br>Prior=Uniform[0.05 ; 0.5]	***Mode***	***Mean***	***Median***	***CI 50%***	***CI 95%***
ABO	0.10	0.24	0.22	0.05-0.50	0.05-0.50
MNSs	0.46	0.33	0.34	0.33-0.48	0.12-0.50
RH	0.09	0.20	0.18	0.05-0.18	0.05-0.44
GM	0.08	0.16	0.12	0.05-0.12	0.05-0.44
mtDNA	0.42	0.29	0.29	0.19-0.45	0.08-0.49
Y-STR	0.11	0.27	0.27	0.05-0.50	0.05-0.50
Y-SNP	0.09	0.24	0.22	0.05-0.23	0.05-0.46
HLA-DRB1(constant selection)	0.18	0.27	0.26	0.13-0.30	0.10-0.48
HLA-DRB1 (different selection in SWE and NWA)	0.20	0.26	0.25	0.13-0.29	0.08-0.47
*Multilocus estimation**	*0.09*	*0.20*	*0.16*	0.06-0.16	*0.05-0.44*
**Selection coefficient *s***<br>Prior=Uniform[0.0 ; 0.15]	***Mode***	***Mean***	***Median***	***CI 50%***	***CI 95%***
ABO	0.000	0.001	0.001	0.000-0.001	0.000-0.003
MNSs	0.002	0.005	0.003	0.000-0.091	0.000-0.091
HLA-DRB1(constant selection)	0.022	0.028	0.026	0.016-0.032	0.007-0.055
HLA-DRB1(Europe)	0.007	0.015	0.011	0.003-0.013	0.000-0.041
HLA-DRB1 (Africa)	0.019	0.032	0.028	0.011-0.034	0.002-0.074

Table S3 Results of ABC estimations under scenario *P*. The values correspond to the weighted posterior distribution [1]. CI=Bayesian Confidence Interval; *Multiloci estimation is done using RH, GM, mtDNA and Y-STR jointly.

5 – Distributions of the statistics used for each of the 7 loci (ABC estimation)

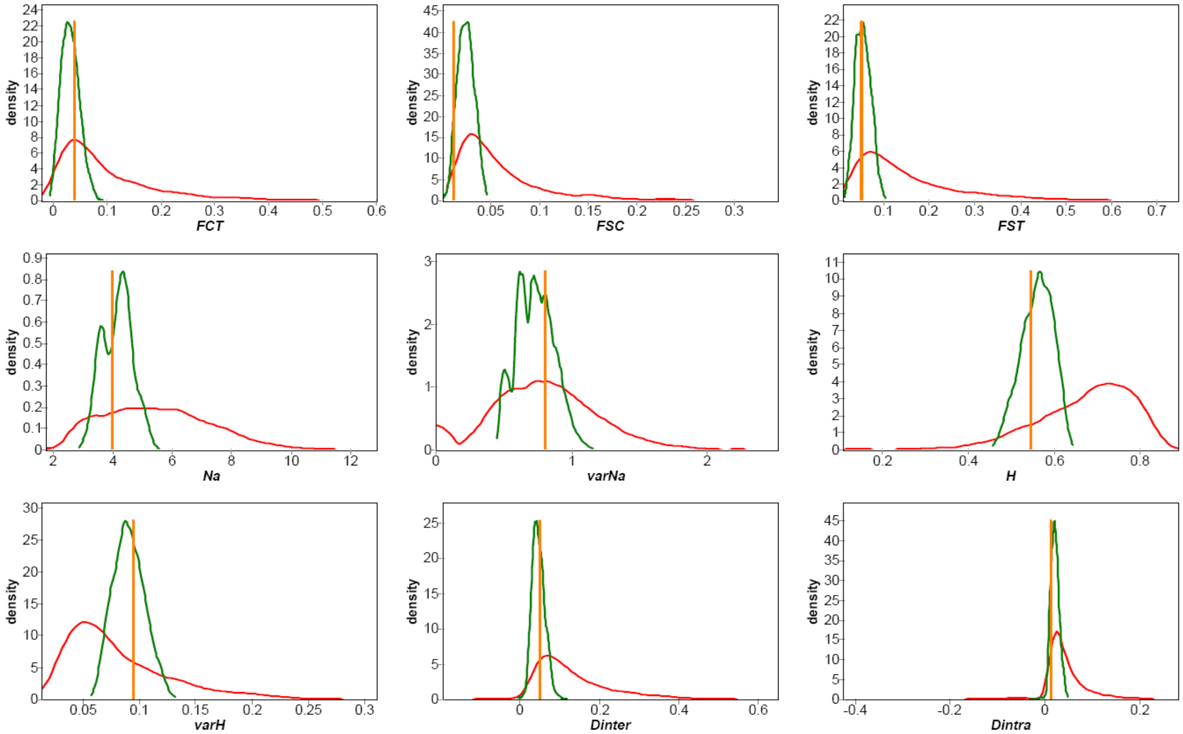

Figure S2 Prior distribution (red), Posterior distribution (green) and observed value (orange bar) for each of the 9 statistics used in the analyses for the locus GM. Based on 1 million simulations and a 0.25% tolerance level.

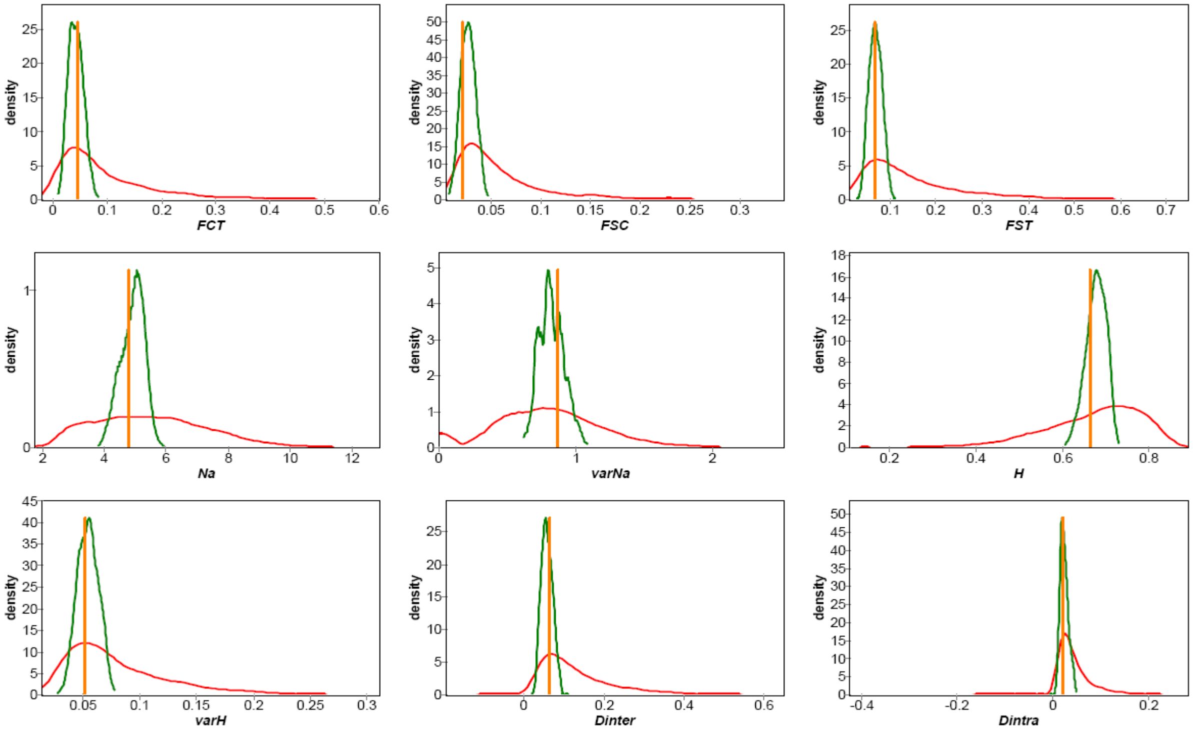

Figure S3 Prior distribution (red), Posterior distribution (green) and observed value (orange bar) for each of the 9 statistics used in the analyses for the locus RH. Based on 1 million simulations and a 0.25% tolerance level.

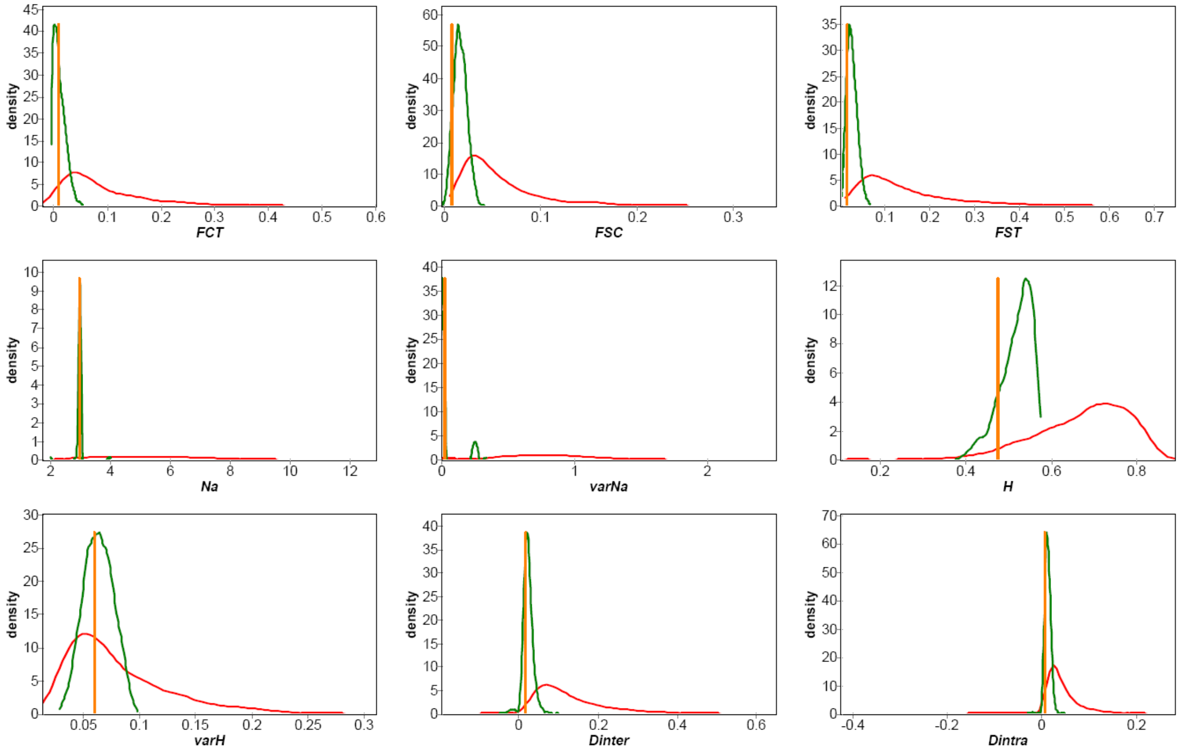

Figure S4 Prior distribution (red), Posterior distribution (green) and observed value (orange bar) for each of the 9 statistics used in the analyses for the locus ABO. Based on 1 million simulations and a 0.25% tolerance level.

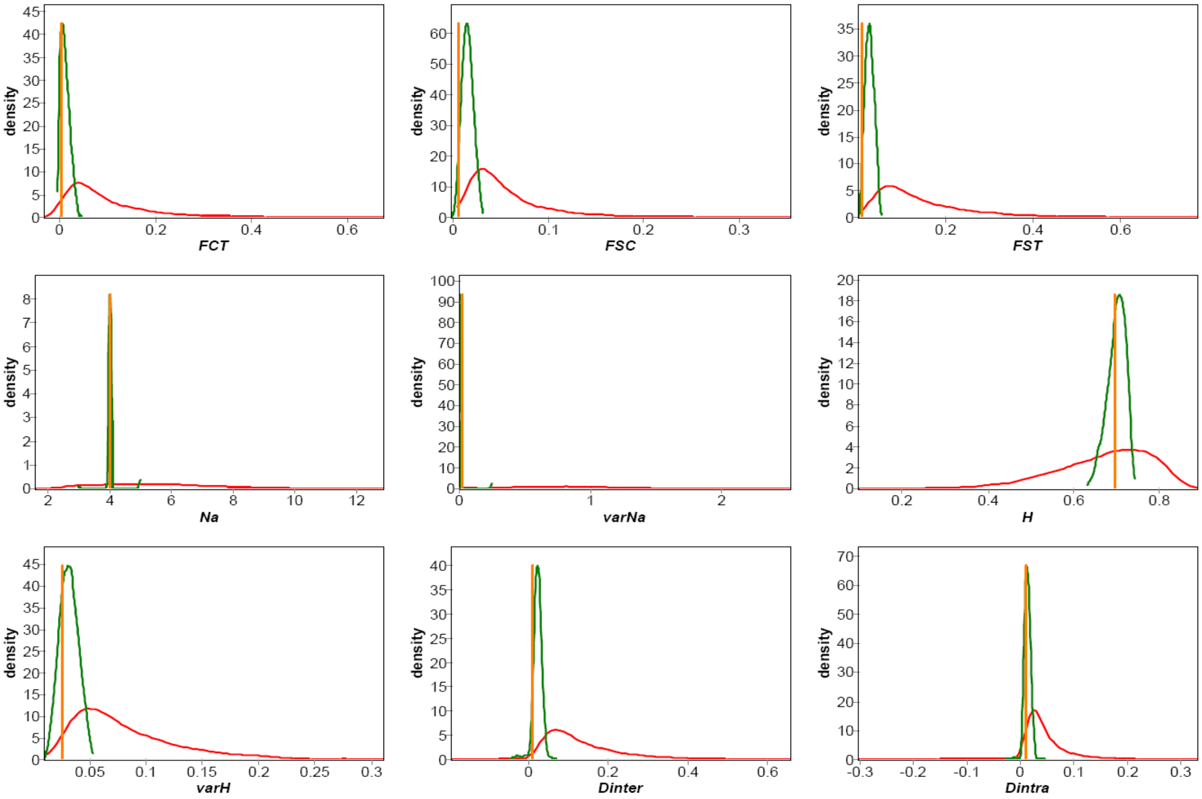

Figure S5 Prior distribution (red), Posterior distribution (green) and observed value (orange bar) for each of the 9 statistics used in the analyses for the locus MNSs. Based on 1 million simulations and a 0.25% tolerance level.

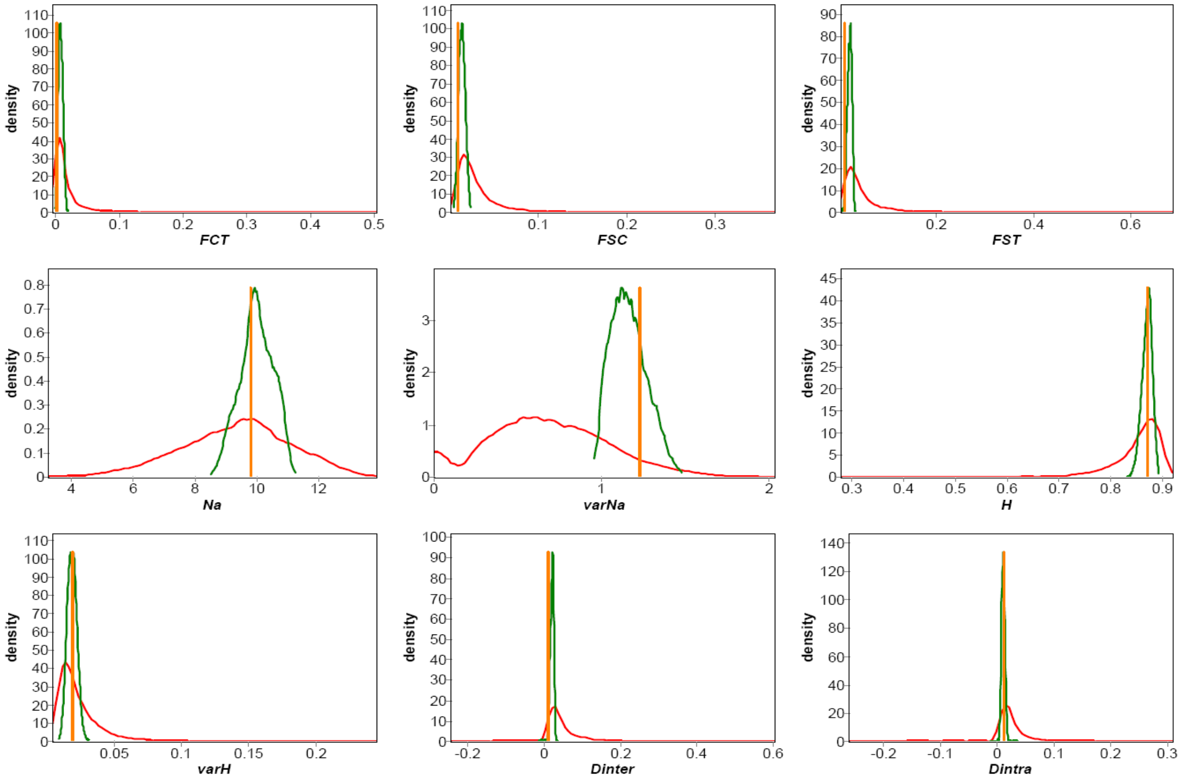

Figure S6 Prior distribution (red), Posterior distribution (green) and observed value (orange bar) for each of the 9 statistics used in the analyses for the locus HLA-DRB1. Based on 1 million simulations and a 0.25% tolerance level.

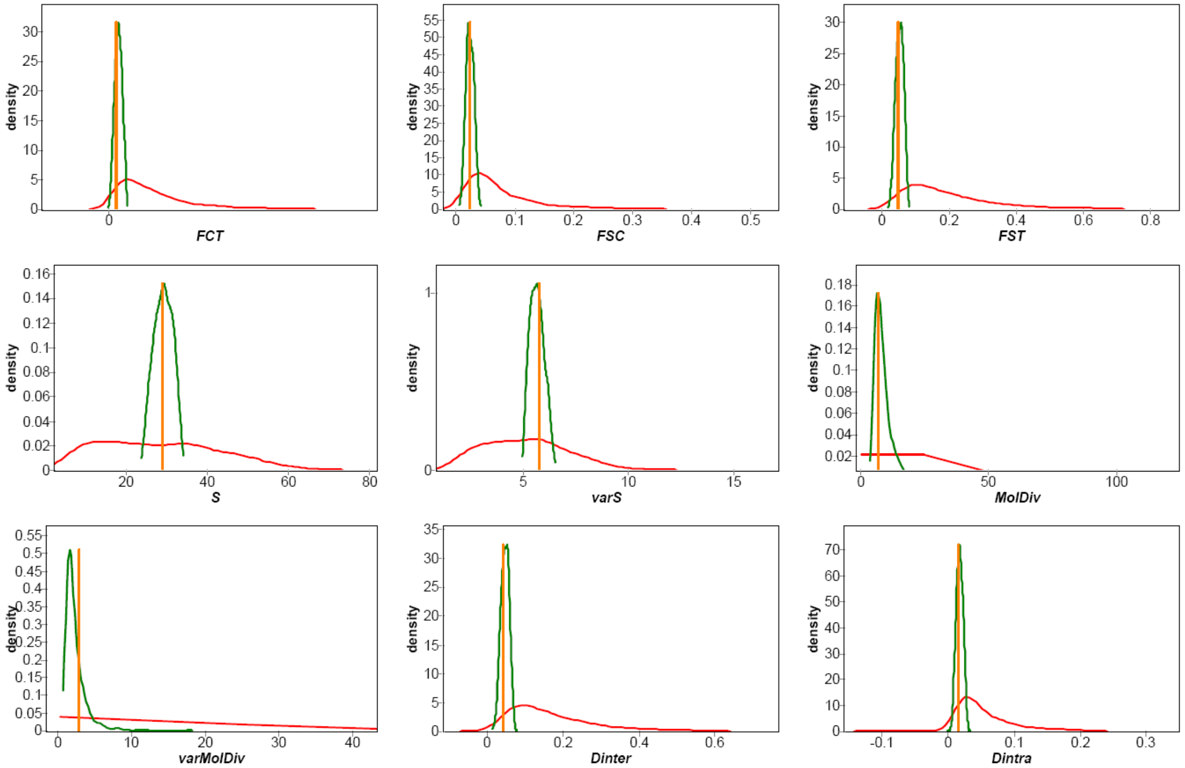

Figure S7 Prior distribution (red), Posterior distribution (green) and observed value (orange bar) for each of the 9 statistics used in the analyses for the mtDNA. Based on 1 million simulations and a 0.25% tolerance level.

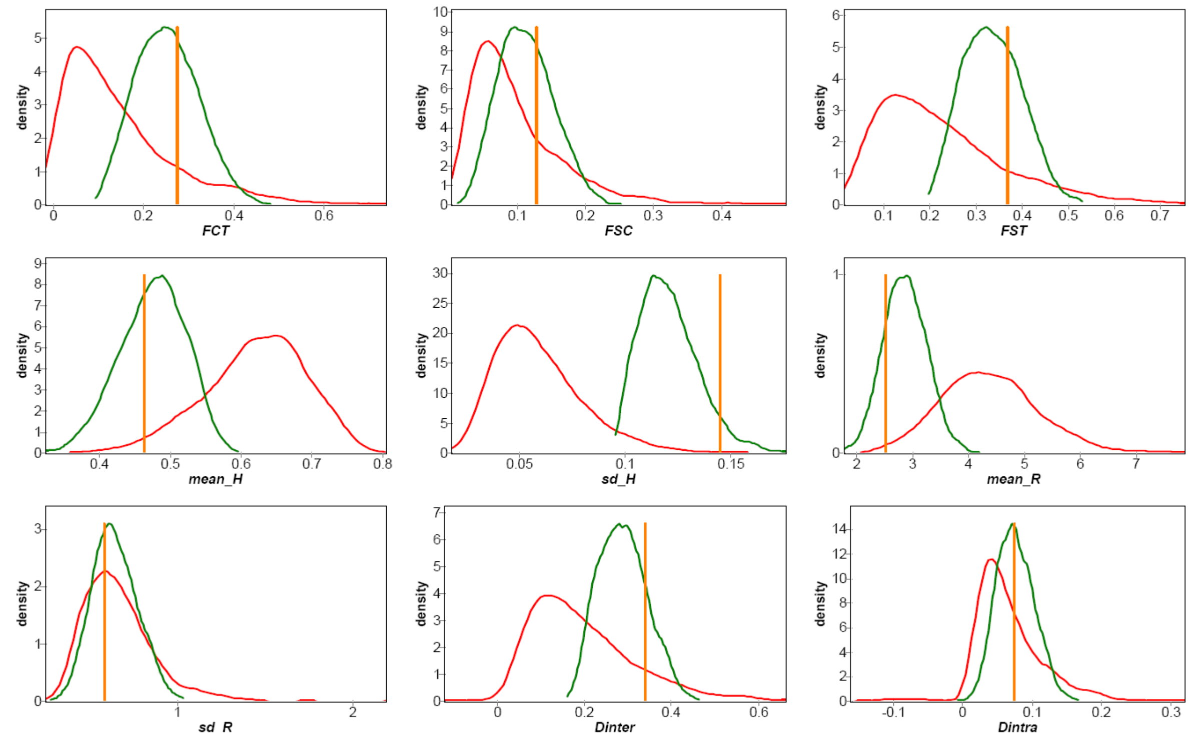

Figure S8 Prior distribution (red), Posterior distribution (green) and observed value (orange bar) for each of the 9 statistics used in the analyses for the Y-chromosome STRs. Based on 1 million simulations and a 0.25% tolerance level.

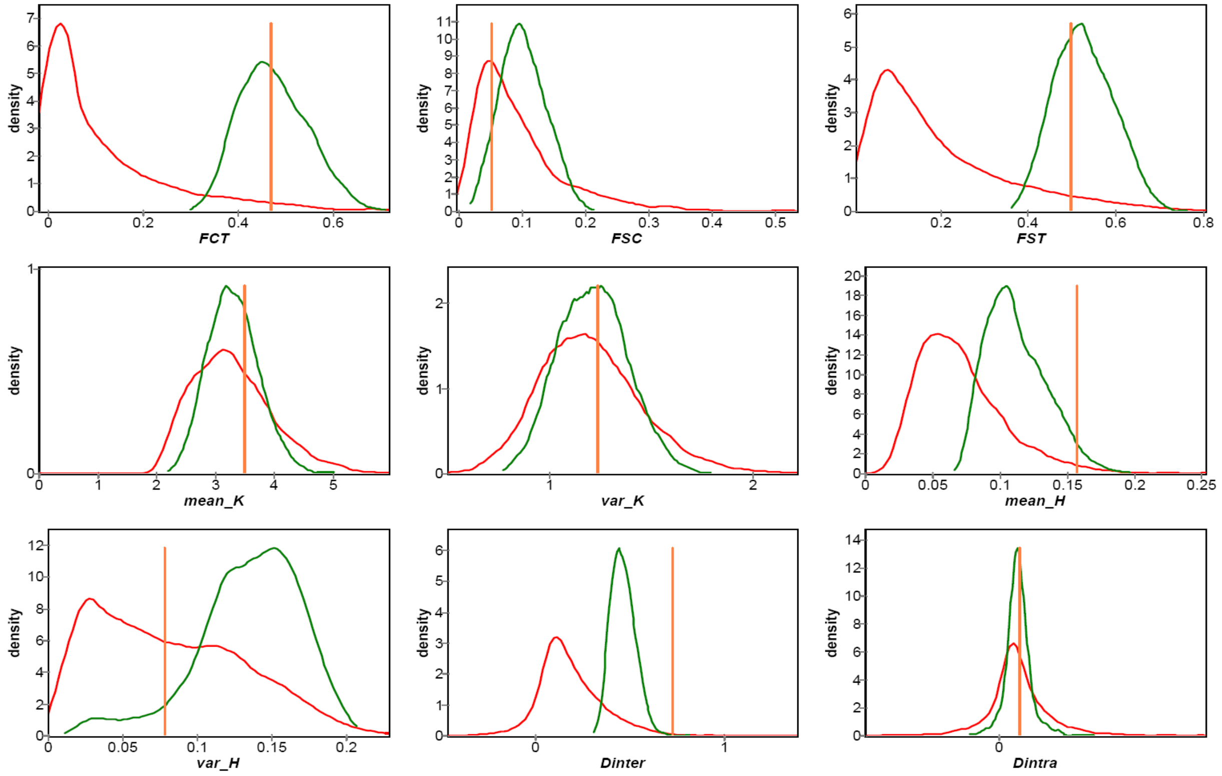

Figure S9 Prior distribution (red), Posterior distribution (green) and observed value (orange bar) for each of the 9 statistics used in the analyses for the Y-chromosome SNPs. Based on 1 million simulations and a 0.25% tolerance level.

6 – Performance evaluation

To test the performance of our approach, we simulated 1,000 pseudo-observed datasets using a fixed set of parameters. Based on this pseudo-observed dataset, but knowing its “true values”, we estimated parameters from the dataset of 1,000,000 simulations. The performance evaluation was an average (over the 1000 pseudo-observed datasets) measurement of the similarity between the “true” and the estimated values. This procedure was carried out on 3 sets of parameters differing by the value of Nm_{inter}: a high value called “weak barrier”, an average value called “middle barrier” and a low value called “strong barrier”. For all pseudo-observed datasets, the values for the other parameters were set to the following values: r = 0.25; Nm_{intra} = 80; *Number of generations* = 800 and coefficient of selection (when applied) s = 0.02. We used an *initial number of alleles* = 10 for the allele frequency test, a *mutation rate* per bp equal to 5×10^{-6} for DNA and an average mutation rate equal to 0.00099 for the STR test.

First, we assessed the potential for a parameter to be correctly estimated in computing the coefficient of determination R^2 [2] which corresponds to the proportion of parameter variance explained by the summary statistics. This information is independent from the posterior distribution of a parameter.

	K	$\boldsymbol{m_{intra}}$	$\boldsymbol{m_{inter}}$	***r***	$\boldsymbol{Nm_{intra}}$	$\boldsymbol{Nm_{inter}}$	***µ****
GM, RH	0.16	0.56	0.37	0.08	0.74	0.51	0.49
mtDNA	0.13	0.51	0.34	0.01	0.68	0.46	0.72
Y-chrom. STRs	0.12	0.48	0.33	0.01	0.66	0.45	0.0005
Y-chrom. SNPs	0.13	0.42	0.28	0.01	0.62	0.41	-°

Table S4 Coefficient of determination (R^2) between the set of statistics and each parameter of the model, computed over 10,000 simulations. * µ corresponds to the number of initial alleles *na* for GM and RH and is an average over the 5 STRs for the Y chromosome. ° no mutation rate is used to generate SNPs as for each SNP a mutation is randomly superimposed on the genealogy. The resulting frequency depends on the position of the mutation on the genealogical tree.

Then, following the strategy of Neuenschwander et al [2], we assessed the quality of the point estimators using the following statistics:

1) Relative bias computed as $bias = \frac{1}{T}\frac{1}{n}\sum_{i=1}^{n}(\theta_i - T)$. A positive relative bias means that the true value is overestimated, respectively underestimated if negative.
2) Relative Root Mean Square Error computed as $RMSE = \frac{1}{T}\sqrt{\frac{1}{n}\sum_{i=1}^{n}(\theta_i - T)^2}$. Because bias and the root RMSE are relative, a value of 1 means a bias (or respectively RMSE) of the size of the true value.
3) Factor 2 statistic [1], which represents the proportion of the estimated values lying in the interval comprised between 50 and 200% of the “true value”. The factor 2 gives information on the absolute precision of the estimator.

4) We also computed the 50%, respectively 90% and 95% coverage (proportion of times in which the true value is within the 50%, respectively 90% and 95% credible interval around the estimate) in order to assess the precision of the Confidence Intervals.

An initial comparison between the Mode, the Mean and the Median of the posterior distribution showed that the Mode is generally the most precise point estimator in our case (result not shown) and we consequently decided to present only the Mode for the next steps of the performance test.

	true value	Mode	Bias	RMSE	Factor-2	50% CI coverage	90% CI coverage	95% CI coverage
	Weak barrier without selection							
r	0.25	0.33	0.30	0.58	0.97	0.52	1.00	1.00
Nm_{intra}	80	80.99	0.01	0.20	1.00	0.58	0.95	0.98
Nm_{inter}	72	35.49	-0.51	0.55	0.30	0.14	0.72	0.86
	Weak barrier with selection							
s	0.02	0.030	0.50	0.67	0.73	0.41	0.86	0.93
r	0.25	0.20	-0.20	0.49	0.51	0.64	1.00	1.00
Nm_{intra}	80	80.01	-0.00	0.24	1.00	0.53	0.93	0.97
Nm_{inter}	72	29.02	-0.60	0.62	0.25	0.03	0.60	0.78
	Middle barrier without selection							
r	0.25	0.32	0.28	0.58	0.93	0.52	1.00	1.00
Nm_{intra}	80	81.10	0.01	0.20	1.00	0.56	0.95	0.98
Nm_{inter}	40	34.97	-0.13	0.41	0.84	0.80	1.00	1.00
	Middle barrier with selection							
s	0.02	0.029	0.44	0.64	0.76	0.48	0.88	0.94
r	0.25	0.20	-0.20	0.49	0.54	0.62	1.00	1.00
Nm_{intra}	80	80.04	0.00	0.24	1.00	0.54	0.94	0.97
Nm_{inter}	40	28.35	-0.29	0.42	0.78	0.68	0.99	1.00
	Strong barrier without selection							
r	0.25	0.28	0.12	0.53	0.88	0.62	1.00	1.00
Nm_{intra}	80	75.99	-0.05	0.21	0.99	0.56	0.95	0.98
Nm_{inter}	8	30.63	2.83	3.37	0.06	0.05	0.73	0.90
	Strong barrier with selection							
s	0.02	0.026	0.28	0.57	0.80	0.53	0.91	0.96
r	0.25	0.14	-0.43	0.51	0.41	0.57	1.00	1.00
Nm_{intra}	80	79.51	-0.01	0.24	0.99	0.53	0.92	0.97
Nm_{inter}	8	24.72	2.09	2.42	0.19	0.59	0.94	1.00

Table S5 Performance test for scenario *P* and allele frequency data.

	true value	Mode	Bias	RMSE	Factor-2	50% CI coverage	90% CI coverage	95% CI coverage
	Weak barrier							
μ	$5x10^{-6}$	$5.47x10^{-6}$	0.09	0.22	1.00	0.96	0.97	0.97
r	0.25	0.26	0.03	0.57	0.69	0.85	1.00	1.00
Nm_{intra}	40	34.40	-0.14	0.30	0.92	0.48	0.89	0.95
Nm_{inter}	36	13.97	-0.61	0.64	0.25	0.07	0.64	0.81
	Middle barrier							
μ	$5x10^{-6}$	$5.42x10^{-6}$	0.08	0.21	1.00	0.97	0.97	0.97
r	0.25	0.25	0.02	0.57	0.66	0.84	1.00	1.00
Nm_{intra}	40	34.60	-0.13	0.29	0.94	0.49	0.91	0.96
Nm_{inter}	20	13.81	-0.31	0.44	0.67	0.66	0.99	1.00
	Strong barrier							
μ	$5x10^{-6}$	$5.3x10^{-6}$	0.06	0.19	1.00	0.98	0.98	0.99
r	0.25	0.19	-0.25	0.57	0.43	0.91	1.00	1.00
Nm_{intra}	40	33.78	-0.16	0.29	0.93	0.51	0.91	0.96
Nm_{inter}	4	11.35	1.84	0.20	1.00	0.98	0.98	0.98

Table S6 Performance test for scenario *P* and 265 bp DNA sequences

	true value	Mode	Bias	RMSE	Factor-2	50% CI coverage	90% CI coverage	95% CI coverage
	Weak barrier							
μ*	0.00099	0.000902	-0.05	0.46	0.76	1.00	1.00	1.00
r	0.25	0.27	0.08	0.56	0.76	0.90	1.00	1.00
Nm_{intra}	40	33.7	-0.16	0.31	0.93	0.53	0.96	0.99
Nm_{inter}	36	12.3	-0.66	0.67	0.14	0.03	0.67	0.87
	Middle barrier							
μ*	0.00099	0.00092	-0.03	0.45	0.78	1.00	1.00	1.00
r	0.25	0.27	0.08	0.56	0.74	0.89	1.00	1.00
Nm_{intra}	40	33.56	-0.16	0.32	0.90	0.53	0.93	0.97
Nm_{inter}	20	12.25	-0.39	0.48	0.58	0.60	0.98	1.00
	Strong barrier							
μ*	0.00099	0.000906	-0.04	0.46	0.77	1.00	1.00	1.00
r	0.25	0.22	-0.11	0.55	0.59	0.92	1.00	1.00
Nm_{intra}	40	33.11	-0.17	0.32	0.91	0.51	0.94	0.98
Nm_{inter}	4	11.01	1.77	2.17	0.30	0.26	0.96	1.00

Table S7 Performance test for scenario *P*. * average over the 5 loci and 5 STRs

	true value	Mode	Bias	RMSE	Factor-2	50% CI coverage	90% CI coverage	95% CI coverage
	Weak barrier							
r	0.25	0.28	0.10	0.50	0.51	0.82	0.93	1.00
Nm_{intra}	40	31.60	-0.21	0.26	0.34	0.86	0.49	0.90
Nm_{inter}	36	11.72	-0.67	0.14	0.69	0.11	0.02	0.59
	Middle barrier							
r	0.25	0.26	0.05	0.51	0.51	0.78	0.92	1.00
Nm_{intra}	40	31.67	-0.21	0.27	0.34	0.86	0.48	0.91
Nm_{inter}	20	11.56	-0.42	0.25	0.49	0.55	0.53	0.97
	Strong barrier							
r	0.25	0.25	-0.01	0.52	0.52	0.92	1.00	1.00
Nm_{intra}	40	31.27	-0.22	0.26	0.34	0.47	0.92	0.97
Nm_{inter}	4	11.06	1.76	1.21	2.14	0.25	0.96	1.00

Table S8 Performance test for scenario *P* and 16 SNPs

	true value	Mode	Bias	RMSE	Factor-2	50% CI coverage	90% CI coverage	95% CI coverage
	Weak barrier							
r	0.25	0.35	0.39	0.63	0.96	0.37	0.97	1.00
Nm_{intra}	80	83.88	0.05	0.17	1.00	0.55	0.93	0.97
Nm_{inter}	72	44.20	-0.39	0.44	0.72	0.23	0.78	0.91
	Middle barrier							
r	0.25	0.35	0.38	0.62	0.97	0.39	0.97	1.00
Nm_{intra}	80	84.45	0.06	0.18	1.00	0.52	0.92	0.97
Nm_{inter}	40	42.13	0.05	0.38	0.88	0.86	1.00	1.00
	Strong barrier							
r	0.25	0.28	0.11	0.54	0.89	0.89	0.48	0.99
Nm_{intra}	80	79.98	0.00	0.15	1.00	1.00	0.58	0.95
Nm_{inter}	8	31.41	2.93	3.43	0.16	0.04	0.62	0.89

Table S9 Performance test for scenario P and 4 combined loci (using STRs for the Y chromosome)

7–Different selection coefficients in Africa and Europe at HLA-DRB1

In all the simulations performed, we made the assumption that balancing selection at HLA-DRB1 was equivalent over all the area under study. In order to evaluate the impact of this assumption on the estimation of the coefficient of selection *s*, we performed 500,000 additional simulations where *s* in NWA and SWE was set independently from two uniform prior distributions [0.0%-2.0%]. In addition to statistics computed as described in the *Material and Method* section (F_{CT}, F_{SC}, F_{ST} and D_{inter}), we also used a series of statistics specific to either NWA or SWE; the mean number of alleles and heterozygosity over samples and their standard deviation in each continent (Na_{Eur}, $var(Na_{Eur})$, H_{Eur}, $var(H_{Eur})$, Na_{Afr}, $var(Na_{Afr})$, H_{Afr}, $var(H_{Afr})$), as well as the mean genetic distance between populations within each continent D_{Eur} and D_{Afr}. Selection coefficients *s* estimated independently in NWA and SWE are presented in Figure 4.

8– Smaller grid

Each scenario was replicated with a smaller grid (noted with *) made up by 64 demes of about 100 x 100 km. Note that we only simulated STRs for the Y chromosome.

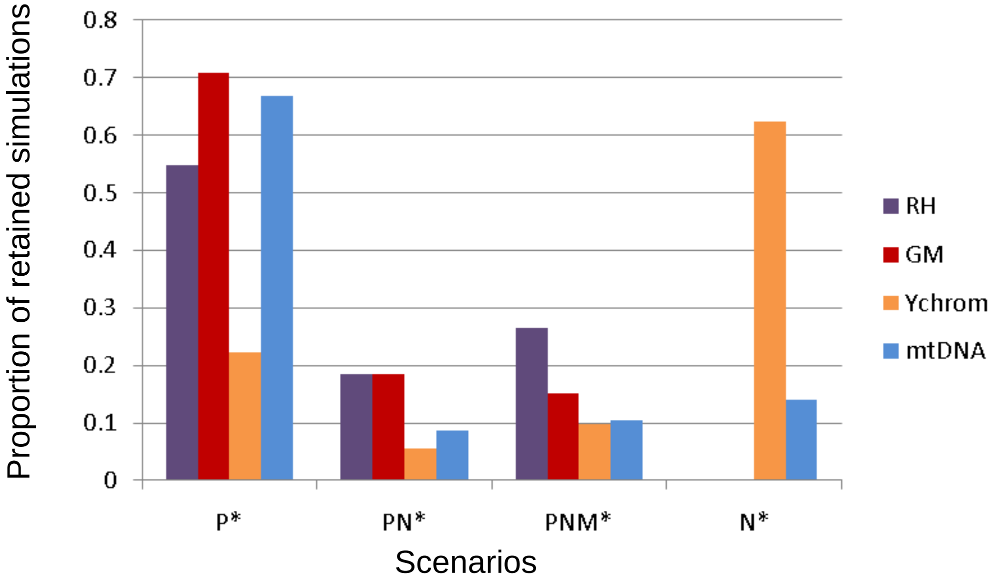

Figure S10 Proportion of the best 0.25% simulations among 80’000 belonging to each of the 4 alternative scenarios, for RH , GM, Y chromosome and mtDNA.

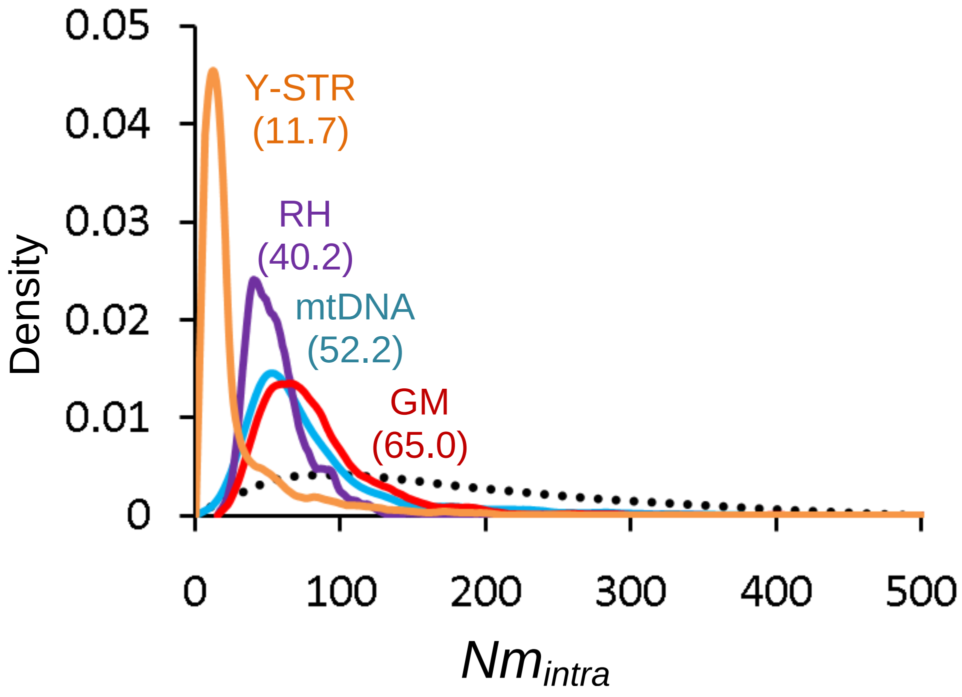

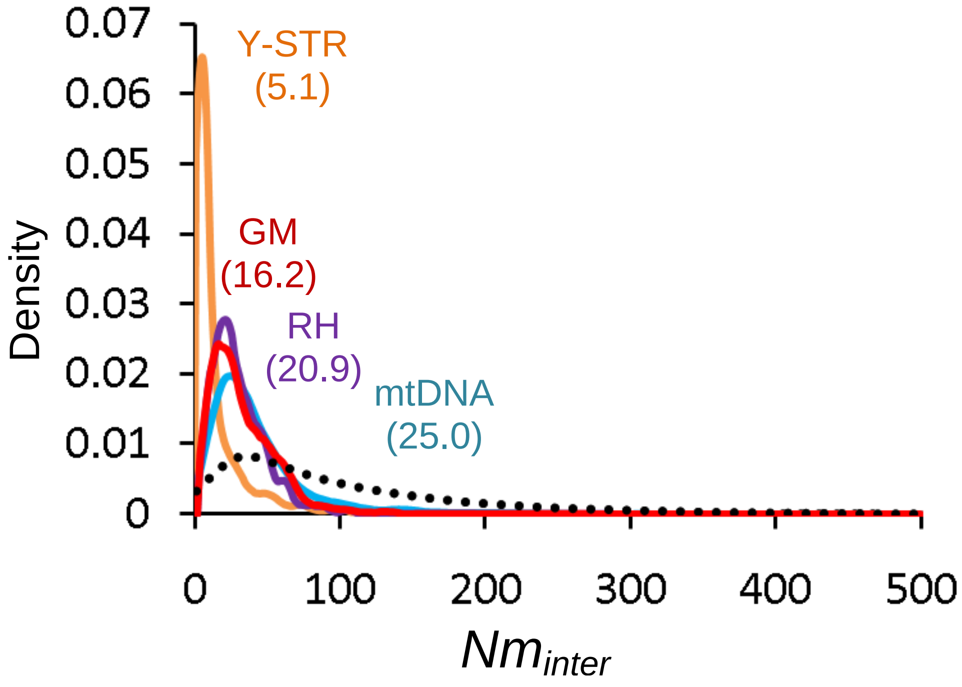

Figure S11 Curves representing the prior (dotted line) and posterior distributions (plain lines) obtained for the parameters of scenario P* and 4 genetic loci: GM, RH, mtDNA, Y-chromosome STRs (Y-STR).

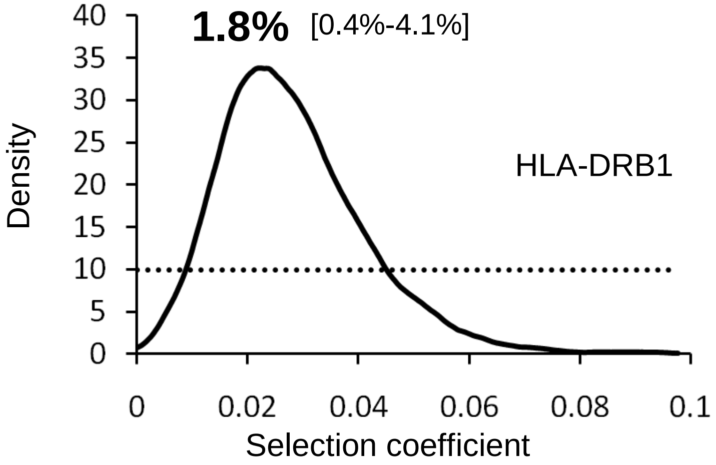

Figure S12 Curves representing the prior (dotted line) and posterior distributions (plain line) obtained for the HLA-DRB1 selection coefficient *s* for scenarios P*. The mode of the posterior distribution is equal to 1.8%.

7 – References

1. Excoffier L, Estoup A, Cornuet JM: Bayesian analysis of an admixture model with mutations and arbitrarily linked markers. *Genetics* 2005, 169:1727-1738.

2. Neuenschwander S, Largiader CR, Ray N, Currat M, Vonlanthen P, Excoffier L: Colonization history of the Swiss Rhine basin by the bullhead (Cottus gobio): inference under a Bayesian spatially explicit framework. *Mol Ecol* 2008, 17(3):757-772.
